# Molecular characterization of infectious bursal disease virus (IBDV) strains of genogroup A2B1 circulating in Delaware, Maryland, and Virginia from 2018 to 2023

**DOI:** 10.1128/spectrum.02976-25

**Published:** 2026-04-16

**Authors:** Sofia Egana-Labrin, Andrew Brodrick, Zubair Khalid, Declan Kehlbeck, Megan Liu, Jimmy Dong, Alex Broadway, Milos Markis, Shankar P. Mondal, Andrew J. Broadbent

**Affiliations:** 1 Department of Animal and Avian Sciences, University of Maryland, College Park, Maryland, USA; 2 Poultry Research and Diagnostic Laboratory, Mississippi State University, College of Veterinary Medicine, Pearl, Mississippi, USA; 3 AviServe LLC, Newark, Delaware, USA; 4 Salisbury Animal Health Laboratory Maryland Department of Agriculture, Salisbury, Maryland, USA; US Food and Drug Administration, Silver Spring, Maryland, USA

**Keywords:** infectious bursal disease virus, IBDV, VP2, VP1, poultry, phylogenetics, reverse genetics

## Abstract

Infectious bursal disease virus (IBDV) causes a major immunosuppressive disease in chickens. In this study, we characterized the IBDV strains circulating in Delaware, Maryland, and Virginia (Delmarva) from 2018 to 2023. The sequence of the hypervariable region (HVR) of the VP2 capsid encoded by segment A, and a region of the VP1 polymerase gene encoded by segment B was obtained from 53 and 34 bursal samples, respectively. Phylogenetic analysis revealed that all the sequences belonged to genogroups A2 and B1, typical of US variant strains. An amino acid signature was identified in the HVR consensus sequence compared with the type-strain Delaware E (Del-E) consisting of amino acid substitutions S215N, S317R, G322E, and E323D. Strains with this signature were previously identified in 2007, in 25% of Delmarva IBDV sequences, and classified as clade two variants. Here, we found this signature had increased in prevalence and was present in 34/53 (64%) of the sequences from 2018 to 2023, including 13/17 (76%) of the sequences from 2023. The signature significantly reduced the virus neutralization titer of serum antibodies raised against Del-E (*P* < 0.05), suggesting that the substitutions could drive immune escape. Additionally, we detected amino acid substitutions in and around a key region of VP1 that correlates with virulence (residues 145–147), suggesting that different IBDV isolates could vary in pathogenic potential. Finally, 2/78 (2.5%) of the IBDV-positive bursal samples were also positive for avian reovirus (ARV), demonstrating that coinfection with multiple immunosuppressive viruses occurs in the field. These findings highlight the importance of ongoing IBDV surveillance.

**IMPORTANCE:**

Infectious bursal disease virus (IBDV) causes a major immunosuppressive disease of poultry. Recently, new strains have emerged and spread in several countries. Ongoing surveillance in the US flocks is therefore critical to determine if any exotic strains of IBDV are circulating. The Delmarva region is a major US poultry-producing area; however, despite its importance, the last reported molecular characterization of IBDV isolated strains was in 2007. Here, we updated the molecular epidemiology of IBDV in the region and uncovered an amino acid signature in the consensus sequence of the capsid hypervariable region comprised of amino acid substitutions S215N, S317R, G322E, and E323D that drives viral escape from neutralizing antibody responses. These findings highlight the importance of ongoing IBDV surveillance and will help to better inform vaccine antigen selection to improve IBD control.

## INTRODUCTION

Infectious bursal disease (IBD) is an immunosuppressive disease of young chickens, resulting in major economic losses to the poultry industry worldwide due to decreased productivity, increased susceptibility to secondary infections, and reduced vaccine efficacy in affected birds (1, 2). The causative agent of IBD is the IBD virus (IBDV), which belongs to the genus *Avibirnavirus* within the family *Birnaviridae*. IBDV is non-enveloped and possesses a bi-segmented double-stranded (ds) RNA genome divided into segment A and segment B. Segment A encodes a polyprotein that is processed into viral protein (VP)2, VP3, and VP4, as well as a nonstructural protein VP5 encoded by an overlapping open reading frame (ORF), while segment B encodes the RNA-dependent RNA polymerase (RDRP), VP1 (1, 3, 4). VP2 forms the capsid and is the main target of vaccine-induced neutralizing antibody responses, and therefore plays a central role in defining viral antigenicity (1, 5). A hypervariable region (HVR) is located within the VP2 gene, spanning amino acid positions 204 to 337, which contains four hydrophilic loops named P_BC_ (amino acids 204–236), P_DE_ (amino acids 240–265), P_FG_ (amino acids 270–293), and P_HI_ (amino acids 305–337), that are thought to be the main antibody binding sites (6–8), and therefore subject to the most intense immune selection pressure (1, 9). Amino acid substitutions in these loops lead to antigenic drift and the emergence of viral variants that can lead to immune escape and vaccine failure (4, 9, 10).

The population of IBDV strains has been classified into several genogroups based on the sequence of the HVR, some with a global distribution and others more restricted to specific geographic regions (2, 5, 11–13). In 2021, Islam et al. developed a classification system where classical virulent strains were grouped as genogroup A1a, while classical attenuated strains fell within A1b. Antigenic variant strains, predominantly found in the United States and China, were assigned to A2, whereas very virulent (vv) strains belonged to A3. Genogroups A4–A8 encompassed more regional lineages, including recombinant and geographically restricted strains from South America, Europe, and Australia (5), and serotype two strains, which do not cause clinical disease in chickens, were grouped as genogroup A0. More recently, Portuguese strains have also been grouped as A9 (12). The sequence of segment B, which encodes VP1, is also critical in defining IBDV diversity. Segment B-based phylogenetics has categorized IBDV into five genogroups (B1–B5) (5), where classical-like strains were grouped B1, vv-like strains were assigned to B2, and early Australian-like, Polish/Tanzanian, and Nigerian strains were classed B3–5, respectively (5). Collectively, the epidemiology of IBDV is therefore described in terms of the A and B genogroups combined, for example, US variant strains belong to genogroup A2B1. The diversity of IBDV strains is expanding globally due to both antigenic drift and reassortment. For example, in the last decade, reassortment between vv A2B3 strains and classical or vaccine A1B1 strains has led to the emergence and spread of novel reassortant A3B1 strains in Europe, and a novel variant (nv) of genogroup A2, known as A2 “distinct” (A2dB1b) has also emerged and rapidly spread through S.E. Asia and Africa, and has recently been found in S. America (1, 14–17).

In the United States, classical (A1B1) strains were first reported in the 1960s, which were initially controlled through the use of live attenuated, inactivated, and autogenous vaccines, but by the mid-1980s, antigenic variant strains (A2B1) had also emerged, complicating disease control due to their altered antigenic profile (2). A2B1 strains are associated with subclinical infection and immunosuppression rather than high mortality; however, they continue to cause significant production losses and welfare issues (5, 9). To date, vv strains have only been sporadically reported in the United States (10, 18, 19), and following an outbreak of vvIBDV, reassortment between vv strains and other endemic US strains has been detected (10). Among the genogroup A2B1 IBDV strains endemic in the United States, different A2 variants have been identified. The “Delaware-E” (Del-E) variant was first isolated in Delaware in the 1980s and is one of the earliest characterized US antigenic variant strains, remaining a prototype for genogroup A2 viruses (2, 5). In addition, the AL-2 variant, isolated from a North Carolina broiler flock by the Allen Lab (University of Delaware), is closely related to Del-E but carries distinct amino acid substitutions in the HVR hydrophilic loops that influence its antigenicity (5, 9).

Given the spread of new distinct variants from S.E. Asia to Africa and S. America, the high prevalence of vvIBDV strains in other countries, and the rise of reassortant strains in Europe, ongoing surveillance in US flocks is critical to determine if any exotic strains are circulating. Moreover, the coexistence of vaccine, classical, variant, and reassortant IBDV strains in US poultry underscores the importance of routine molecular surveillance in different poultry producing regions, and the use of vaccines tailored to the antigenic profile of circulating field strains (1, 2, 10).

The Delmarva region of the USA (Delaware, Maryland, and Virginia) is a major poultry-producing area. In 2024, it raised 613 million chickens and generated $4.7 billion in wholesale value. However, despite the importance to the US poultry industry, the last molecular characterization of IBDV strains circulating in the region isolated strains in 2007 (20). At the time, five clades of IBDV were found to have evolved from the Del-E strain, based on the presence of amino acid substitutions in the HVR. Moreover, clade five was found to break through vaccine-induced protection in challenged birds, suggesting some of the HVR substitutions contributed to immune escape (20). In the present study, we aimed to update the molecular epidemiology of IBDV in the Delmarva region to determine how the clades have evolved since the last published study.

## MATERIALS AND METHODS

### Sampling protocol

Bursal samples (*n* = 120) were retrieved between 2018 and 2023 from Delaware, Maryland, and Virginia (Delmarva) broiler farms through collaborative molecular surveillance between the University of Maryland (UMD), the Salisbury Animal Health Laboratory at the Maryland Department of Agriculture, and AviServe LLC in Delaware. Samples were collected by poultry veterinarians from sentinel birds as part of routine surveillance, or from commercial birds because of a suspicion of an IBDV infection based on clinical presentation and the existence of potential concomitant or secondary infections. Samples were also submitted for molecular characterization based on the presence of macroscopic bursal lesions such as swelling or atrophy and lymphoid depletion of the bursa (Table S1). Bursal samples belonging to the same farm were pooled and classed as one sample. Samples were shipped to UMD on dry ice, whereupon they were stored at −80°C until processing in the laboratory.

### Bursa homogenization, RNA extraction, reverse transcription polymerase chain reaction (RT-PCR), and Sanger sequencing of the VP2 HVR and partial VP1 gene

Briefly, 20 mg of pooled bursal tissue from one farm was placed into a lysing matrix D tube with ceramic spheres and homogenized using the FastPrep-24 lysis system (MP Biomedicals, Santa Ana, CA), according to the manufacturer protocols and stored at −80°C until further use. RNA was extracted from the bursal homogenates, using QIAshredder and RNeasy kits (Qiagen, Valencia, CA) according to the manufacturer’s instructions. Complementary DNA (cDNA) was generated using SuperScript III Reverse Transcriptase (Invitrogen, Carlsbad, CA) and a random primer (IDT, Coralville, IA). A 579 base pair (bp) fragment of the VP2 gene that included the HVR was amplified using primers described by (2): 743-F (5′-GCCCAGAGTCTACACCAT-3′) and 1331-R (5′-ATGGCTCCTGGGTCAAATCG-3′ (2), and a 722 bp fragment of the VP1 gene was amplified using primers described by reference 10: B-168A-F (5′-CATAAAGCCTACAGCTGGAC-3′) and B-889-R (5′-GTCCACTTGATGACTTGAGG-3′) (10). The amplification of both regions was performed using DreamTaq Green PCR kit (Thermo Fisher Scientific, Waltham, MA) and the same reaction conditions described previously (2). Briefly, after an initial denaturation at 94°C for 2 min, DNA was amplified through 35 cycles, each consisting of denaturation at 95°C for 30 s, annealing at 57°C for 1.5 min, and extension at 72°C for 1.5 min, followed by a final extension at 72°C for 5 min. The PCR products were then separated by electrophoresis through a 1% agarose gel with tris-borate EDTA (TBE) buffer (Thermo Fisher Scientific, Waltham, MA). The resulting bands were excised and purified using the Monarch Genomic DNA Purification Kit (New England Biolabs, Ipswich, MA), and the DNA submitted for Sanger sequencing (Azenta Life Sciences, Burlington, MA). Sequences were assembled, aligned, and analyzed using Geneious Prime, version 2025.0.3 (Biomatters, Auckland, New Zealand) (21) and uploaded to GenBank, with the following Accession numbers: PX233970–PX234007 (HVR VP2, Segment A), and PX234023–PX234051 (Partial VP1, Segment B).

### PCR amplification and MinION sequencing of VP2 and VP1 sequences

To amplify larger regions of the genome, a 1,827 bp region was amplified, targeting the whole VP2 coding sequence (1,536 bp), using a set of in-house primers: A-42-F (5′-CAGGATGGAACTCCTCCTTCT-3′) and A-1869-R (5′-GCTGTTCAGTGCTTTGGGTG-3′). The amplification was performed with DreamTaq Green PCR Master Mix kit (Thermo Fisher Scientific, Waltham, MA). Briefly, after an initial denaturation at 95°C for 3 min, DNA was amplified through 35 cycles, each consisting of denaturation at 95°C for 30 s, annealing at 54°C for 30 s, and extension at 72°C for 2 min, followed by a final extension at 72°C for 10 min. The 2,704 bp coding region of VP1 was amplified using a set of in-house primers: B-50-F (5′-CACGTTAGTGGCTCCTCTTCT-3′) and B-2754-R (5′-TCCTCTTCTTGAGTGGTTCC-3′). The amplification was performed with DreamTaq Green PCR Master Mix kit (Thermo Fisher Scientific, Waltham, MA). Briefly, after an initial denaturation at 95°C for 3 min, DNA was amplified through 35 cycles, each consisting of denaturation at 95°C for 30 s, annealing at 56°C for 30 s, and extension at 72°C for 4 min, followed by a final extension at 72°C for 10 min. In both cases, the PCR products were separated by electrophoresis in a 1% agarose gel with TBE buffer. The resulting bands were excised and purified using the Monarch Genomic DNA Purification Kit, and the DNA was submitted for sequencing by Oxford Nanopore (MinION, Oxford, UK) Technology (Plasmidosaurus, San Francisco, CA). Sequences were assembled, aligned, and analyzed using 213 (Biomatters, Auckland, New Zealand) (21) and uploaded to GenBank, with the following Accession numbers: PX234008–PX234022 (whole mature VP2 peptide, Segment A), and PX233332–PX233336 (whole VP1, Segment B).

### Identification of other viral pathogens in the samples

After RNA extraction, complementary DNA (cDNA) was synthesized using SuperScript III Reverse Transcriptase (Invitrogen, Waltham, MA) with a random primer mix, and subject to RT- PCR using published primers and protocols targeting avian reovirus (22), avian rotavirus (23), and chicken astrovirus (23). The amplification of each sample was conducted using DreamTaq Green PCR kit (Thermo Fisher Scientific, Waltham, MA). Amplicons were visualized using gel electrophoresis.

### Phylogenetic and evolutionary analyses

The HVR sequences (Segment A), and the partial VP1 sequences (Segment B) were aligned with VP2 and VP1 genotyping reference sequences (5), and a multiple sequence alignment was performed using a Clustal Omega plugin in the Geneious Prime Software. Phylogenetic trees were constructed using the maximum likelihood method with the GTR GAMMA nucleotide model and 1,000 bootstrap replications, using the Geneious Prime Software. Bootstrap values lower than 75% were considered non-significant. The phylogenetic tree constructed for the HVRs contained the sequences of 53 Delmarva samples and 93 reference sequences published in GenBank (5), and the sequences in the alignment were all trimmed to 293 nucleotides (nt) to be the same length, spanning nucleotides 764–1,255 (amino acids 212 to 375) in the VP2 HVR coding region. The phylogenetic tree constructed for the partial VP1 contained the sequences of 34 Delmarva samples and 78 reference sequences (5), and the sequences in the alignment were trimmed to 166 nucleotides to be the same length, spanning nucleotides 328–825 (amino acids 73 to 238) in the Segment B partial VP1 coding region. The generated phylogenetic trees were visualized using the Interactive Tree of Life Tool (iTOL) (24). To assess temporal evolutionary dynamics, phylogenetic analysis of the VP2 hypervariable region (HVR) was performed using the maximum likelihood (ML) method implemented in MEGA12 (25). The Tamura–Nei nucleotide substitution model (26) was applied, and the tree with the highest log likelihood was selected. The initial tree for the heuristic search was chosen based on the higher log likelihood between Neighbor-Joining (NJ) (27) and Maximum Parsimony (MP) trees. The temporal signal and clock-likeness of the VP2 HVR nucleotide sequences were assessed using root-to-tip regression analysis implemented in TempEst v1.5.3 (28). The ML tree generated in MEGA12 and the collection year of each strain were used as input. Root-to-tip genetic distances were plotted against sampling year to evaluate the correlation between genetic divergence and time. The best-fitting root was determined by minimizing the sum of squared residuals and maximizing the correlation between root-to-tip divergence and sampling year. Linear regression analysis within TempEst was used to estimate the strength of the temporal signal.

The data set comprised 53 HVR sequences generated in this study and 17 US genogroup A2 reference strains retrieved from GenBank. All sequences were trimmed to nucleotides 649–1,125 of VP2, resulting in 477 aligned nucleotide positions included in the final analysis.

### Amino acid alignments

The nucleotide sequences were translated into amino acid sequences using a Clustal Omega 1.2.2 plugin in the Geneious Prime Software. For the HVR alignment, the amino acid sequences were truncated to 164 residues in length, from amino acids 212 to 375 in the VP2 encoding gene. For the partial VP1 alignment, the amino acid sequences were truncated to 166 residues in length, from amino acids 73 to 238. For the whole VP2 precursor peptide and whole VP1 sequence alignments, the sequences were trimmed to 512 and 879 amino acids, respectively. In all the alignments, amino acid sequences corresponding to IBDV strains Del-E and AL-2 were used as comparisons. The percentage of samples with each amino acid substitution was plotted using GraphPad Prism (29). The amino acid variability within the VP2 hypervariable region (HVR) of the recent Delmarva isolates was compared to the Delaware E and AL-2 strains and visualized using sequence logo analysis (30, 31).

### Structural modeling

The structure of the consensus sequence of the HVR trimer was modeled using a modified version of AlphaFold v3 (32). The predicted structure was generated by adding the substitutions S254N, S317R, G322E, and E323D to the Del-E amino acid sequence *in silico*. The resultant prediction was then downloaded and processed using PyMol (v2.5; Schrödinger) to visualize the structure and highlight amino acid substitution sites. The prediction was then aligned to a published VP2 trimer extracted from an IBDV capsid structure solved by Cryo-EM (8), obtained from the Research Collaboratory for Structural Bioinformatics (RCSB) Protein Data Bank (accession code 7VRN), to determine how reasonable the model was. The alignments showed agreement between the structures (33) (root mean square deviation [RMSD] = 1.27 Å). The structure of the VP1 polymerase was modeled using a modified AlphaFold v3, and the resultant prediction was processed using PyMol to visualize the structure and highlight amino acid substitution sites.

### Cell lines and antibodies

The chicken B-cell lymphoma line DT40 (ATCC, catalog number CRL-2111) was cultured in RPMI medium supplemented with 10% heat-inactivated fetal bovine serum (FBS) (Sigma-Aldrich, St. Louis, MO), 1% L-glutamine (Sigma-Aldrich, St. Louis, MO), 10% Tryptose phosphate broth (Sigma-Aldrich), 1 mM Sodium pyruvate (Sigma-Aldrich, St. Louis, MO), and 50 mM Beta-mercaptoethanol (Gibco, Thermo Fisher Scientific, Waltham, MA). Cells were cultured at 37°C in an atmosphere of 5% CO_2_. For immunostaining, a mouse monoclonal antibody targeting the IBDV VP3 protein (clone FC3) was used as the primary antibody (3, 34). As a secondary antibody, we employed a goat anti-mouse monoclonal antibody conjugated to Alexa Fluor 488 (Invitrogen, Thermo Fisher Scientific).

### Generation of chimeric viruses containing the HVR from Del-E and a contemporary Delmarva variant strain

The complete HVR sequences of the Del-E strain and a strain containing amino acid substitutions S254N, S317R, G322E, and E323D (“NRED”) were constructed by reverse genetics. Briefly, the reverse genetics plasmid encoding segment A from laboratory-adapted IBDV strain PBG98 (pPBG98A [3]) was used as a backbone, into which the HVR from Del-E or NRED was inserted. The plasmids were synthesized (GenScript, Piscataway, NJ) and cloned into a pSF-CAG-KAN expression vector (Addgene, Watertown, MA) using competent DH5-alpha *E. coli* (New England Biolabs, Ipswich, MA) (4). The resulting chimeric plasmids were purified using a plasmid kit (Qiagen, Valencia, CA) according to the manufacturer’s protocol and submitted for Oxford Nanopore Technology sequencing (Plasmidosaurus, San Francisco, CA) to confirm their identity. These plasmids were combined with the reverse genetics plasmid encoding segment B (pPBG98B [3]) to rescue viruses PBG98-HVR^DEL-E^ and PBG98-HVR^NRED^. Briefly, DT40 cells were resuspended in 100 µL Opti-MEM medium to contain a total of 1 × 10^7^ cells. For each chimera, 10 µg of segment A and 10 µg of segment B plasmids were mixed with the resuspended DT40 cells in 2-mm electroporation cuvettes (BTX). The mixture was then electroporated at 225 V and a pulse width of 2 ms of poring pulse. The cells were placed into a six-well plate and incubated at 37°C with 5% CO_2_. Forty-eight hours post-electroporation and every 72 h thereafter, fresh DT40 cells were added to the old cultures in a ratio of 3:1. Passages were limited to five times. Virus rescue was confirmed by immunofluorescence microscopy (IFM), as previously described (4). The sequences of the HVRs of the rescued chimeric viruses were confirmed by using Oxford Nanopore Technology sequencing (Plasmidosaurus, San Francisco, CA).

### Quantification of viral replication kinetics

DT40 cells were infected with the PBG98-HVR^DEL-E^ and PBG98-HVR^NRED^ viruses at a multiplicity of infection (MOI) of 0.01 in U-bottomed 96 well-plates (Thermo Fisher Scientific). Cells were inoculated for 1 h at 37°C and then washed and incubated. At 24, 48, and 72 hours post-infection (hpi), RNA was extracted using an RNeasy kit (Qiagen), as per the manufacturer’s instructions. The RNA was reverse transcribed, and IBDV was amplified by quantitative polymerase chain reaction, as previously described (35). The fold change in viral genome expression was determined by normalization to a housekeeping gene (RPLPO) and expressed relative to mock-infected cells in a ΔΔCt analysis, as previously described (35).

### Virus cross-neutralization assessment of the rescued viral chimeras

To evaluate the antigenic relatedness of the PBG98-HVR^DEL-E^ and PBG98-HVR^NRED^ viruses, a cross-neutralization assay was designed. First, chimeric virus stocks were titrated in DT40 cells using the Reed and Muench method and expressed as the 50% tissue culture infectious dose (TCID₅₀)/mL (13). The endpoint was determined by fluorescence microscopy, as previously described (36). Next, serum was obtained from chickens that had been hyperimmunized against variant E (Del-E) (AVSBio, Norwich, CT), heated at 56°C for 30 min, and then serially diluted twofold from 1:640 to 1:1,310,720 in DT40 medium in 96-well plates. The diluted serum was then incubated with 10^3^ TCID₅₀/mL of each of the chimeric viruses in triplicate for 1 h at 37°C. Following incubation, the serum-virus mixtures were added to 8 × 10^4^ DT40 cells per well in 96-well U-bottom plates and incubated for 72 h at 37°C with 5% CO_2_. To calculate the virus neutralization (VN) titers, the DT40 cells were fixed and stained according to a previously described protocol (4). Briefly, cells were fixed with 4% paraformaldehyde (Sigma-Aldrich, St. Louis, MO) for 20 min, permeabilized with 0.1% Triton X-100 (Sigma-Aldrich, St. Louis, MO) for 10 min, and blocked with 4% BSA for 60 min. Immunofluorescence detection was performed by incubating the cells with an IBDV VP3 protein primary mouse monoclonal antibody (4) at room temperature for 1 h. After washing with PBS, the cells were incubated in the dark for 1 h with a goat anti-mouse secondary antibody conjugated to Alexa Fluor 488 (Thermo Fisher Scientific, Waltham, MA). This was followed by a final wash and a 10-min incubation with 4′,6-diamidino-2-phenylindole (DAPI) (Invitrogen, Thermo Fisher Scientific) for nuclear staining. Imaging was performed using an inverted fluorescence microscope (Axio Zeiss). The VN titer was determined as the log₂ of the highest serum dilution at which no VP3-positive cells were detected.

## RESULTS

### Bursal samples were obtained from 2018 to 2023 in Delmarva

A total of 120 bursal samples were obtained from 92 commercial farms and one backyard flock in Delmarva, with 33 samples originating from Delaware (DE), 28 from Maryland (MD), 12 from Virginia (VA), and 47 were from the Delmarva region, but the state was not disclosed (Table S1). One sample was from 2018, 11 were from 2019, 17 were from 2020, 56 were from 2021, 7 were from 2022, and 28 were from 2023. A total of 68 samples were from sentinel chickens, 52 were from commercial chickens, and 1 sample was obtained from a backyard chicken. Samples were obtained from chickens at 15 to 36 days of age, except for the backyard chicken which was 9 months of age. Of the non-sentinel birds, 49 had been clinically characterized: the daily mortality reported on these farms ranged between 0.18 to 1.2%, and 39/49 (79.6%) bursal samples had macroscopic lesions/abnormalities, such as enlargement (indicative of inflammation and edema). Of the 39 samples with macroscopic changes, histopathological assessment revealed moderate (8/39), mild-to-moderate (4/39), and marked (24/39) diffuse to multifocal follicular lymphoid depletion, consistent with IBDV infection, and 3 samples did not have a histopathological report (Table S1). IBDV was confirmed by RT-PCR targeting either the HVR or a region of the VP1 gene in 78 (65%) of the submitted samples (Table 1; Table S1). Of the 78 IBDV-positive samples, ARV was also confirmed in 2 (2.6%), demonstrating that co-infection was observed in the field (Table 1; Table S1). Avian rotavirus and chicken astrovirus were not detected.

**TABLE 1:** Information on the IBDV-positive cases from Delmarva 2018–2023 from which sequences were obtained^*a*^

				Segment A			Segment B		
Sample ID	Year	State	Bursal lesions	RT-PCR HVR	Sequence HVR	Sequence whole VP2	RT-PCR VP1	Sequence partial VP1	Sequence whole VP1
D1	2018	Delaware	na	+	+	+	+	+	–
D2	2019	Delaware	na	+	+	+	–	–	–
D3	2019	Delaware	na	+	+	+	+	+	–
D8	2019	Delmarva	na	+	–	–	+	+	–
D11	2019	Delmarva	na	+	+	+	+	+	–
D15	2020	Delmarva	na	+	+	–	+	+	–
D17	2020	Delmarva	na	+	+	–	+	+	–
D18	2020	Delmarva	na	+	+	+	+	+	–
D21	2020	Delmarva	na	+	+	+	–	–	–
D22	2020	Delmarva	na	+	+	–	+	+	–
D25	2020	Delmarva	na	+	+	–	+	+	–
D27	2020	Delaware	na	+	+	–	+	+	–
D29	2020	Delaware	na	+	–	–	+	+	–
M7	2021	Virginia	+	+	+	+	+	+	–
M8	2021	Maryland	+	+	+	–	+	+	–
M11	2021	Maryland	+	+	+	–	+	–	–
M15	2021	Delaware	+	+	+	–	+	+	–
M19	2021	Maryland	+	+	+	–	+	+	–
M23	2021	Maryland	+	+	+	+	+	–	–
M25	2021	Virginia	na	+	+	+	–	–	–
D48	2021	Delaware	na	+	+	+	+	+	+
D49	2021	Delaware	na	+	+	–	+	+	–
D55	2021	Delmarva	na	+	+	+	+	+	+
D56	2021	Delmarva	na	+	+	–	+	+	–
D58	2021	Delmarva	na	+	+	–	–	–	–
D60	2021	Delmarva	na	+	+	–	+	+	–
D62	2021	Delmarva	na	+	+	–	+	+	–
D63	2021	Delmarva	na	+	+	–	+	+	–
D67	2021	Delmarva	na	+	+	–	–	–	–
D70	2021	Delaware	na	+	+	–	–	–	–
D72	2021	Delaware	na	+	+	–	–	–	–
D75	2021	Delmarva	na	+	+	–	–	–	–
D76	2021	Delmarva	na	+	+	+	+	+	+
D77	2021	Delmarva	na	+	+	–	–	–	–
D78	2021	Delmarva	na	+	+	–	–	–	–
M28	2022	Maryland	na	+	+	–	+	+	–
D88	2022	Delmarva	na	+	+	–	–	–	–
D89	2022	Delmarva	na	+	+	–	–	–	–
M30	2023	Delaware	+	+	+	–	+	+	–
M31	2023	Maryland	+	+	+	+	+	+	–
M32	2023	Maryland	+	+	+	–	+	+	–
M33	2023	Virginia	+	+	+	–	–	–	–
M35	2023	Virginia	+	+	+	–	+	–	–
M36	2023	Virginia	+	+	+	–	+	–	–
M38	2023	Maryland	+	+	+	–	–	–	–
M39	2023	Maryland	+	+	+	–	–	–	–
M42	2023	Maryland	+	+	+	–	–	–	–
M50	2023	Virginia	na	+	+	–	+	–	–
D91	2023	Delmarva	na	+	+	–	+	+	–
D92	2023	Delmarva	na	+	+	–	+	+	–
D93	2023	Delmarva	na	+	+	+	+	+	+
D94	2023	Delmarva	na	+	+	–	+	+	–
D95	2023	Delmarva	na	+	+	+	+	+	+
D96	2023	Delmarva	na	+	+	–	+	+	–
D97	2023	Delmarva	na	+	+	–	+	+	–

^*a*^ na, information not available; “+” Positive, “–” negative.

### All IBDV sequences belonged to genotypes A2 and B1

**Fig 1:**
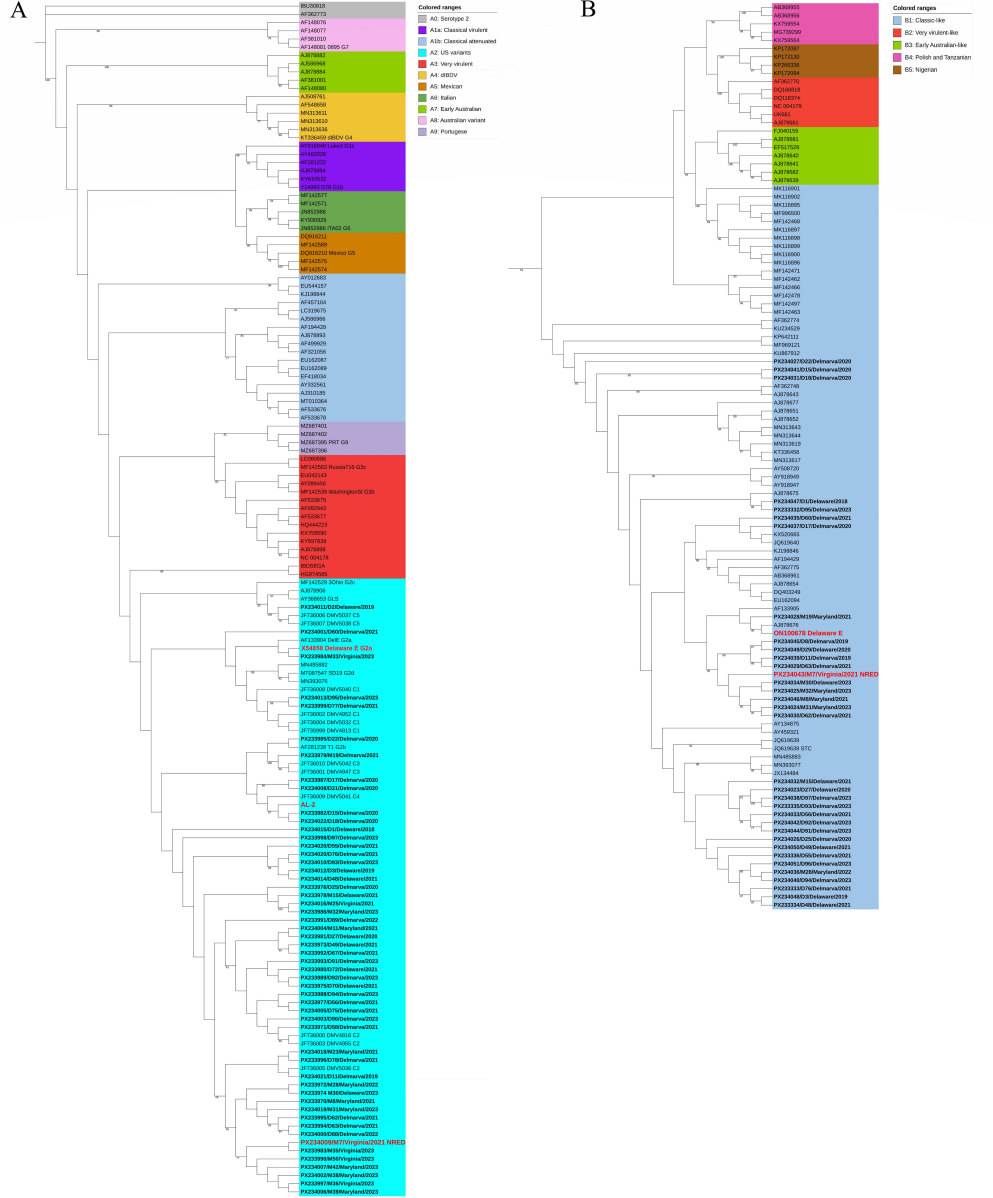
Phylogenetic trees based on the VP2 HVR, encoded by Segment A, and a region of the VP1 gene, encoded by Segment B. The phylogenetic trees were constructed with the genetic sequences of the strains obtained from the Delmarva region between 2018 and 2023. Each sequence was represented by the GenBank accession number, strain name, location, and year of detection, in bold. The trees were constructed and visualized using the maximum likelihood method with the GTR GAMMA nucleotide model and 1,000 bootstrap replications, using the Geneious Prime software. The phylogenetic data were visualized using the interactive tree of life tool (iTOL). Bootstrap values lower than 75% were considered as non-significant. The reference strains Delaware E and AL-2, commonly used as vaccines, and the strain characterized in this study (M7/Virginia/2021) were colored in red. (**A**) The tree was constructed using 53 HVR nucleotide sequences obtained from Sanger sequencing of the PCR products (GenBank accession numbers PX233970–PX234022) and the nucleotide sequences of 91 reference IBDV strains in GenBank (indicated by the remaining accession numbers). All the sequences were trimmed to nucleotides 764–1,255 of the VP2 gene, to be the same length. The tree was divided into 11 sections, each depicted in a different color and representing genogroups A1a and A1b-9 within Serotype 1, as well as Serotype 2 in gray, as indicated by the key. (**B**) The tree was constructed using 34 partial VP1 nucleotide sequences obtained from Sanger sequencing of the PCR products (GenBank accession numbers PX234023–PX234051 and PX233332–PX233336) and the nucleotide sequences of 74 reference IBDV strains in GenBank (indicated by the remaining accession numbers). All the sequences were trimmed to nucleotides 328–825 of the VP1 gene, to be the same length. The tree was divided into five sections, each depicted in a different color and representing genogroups B1-5, as indicated by the key.

**Fig 2:**
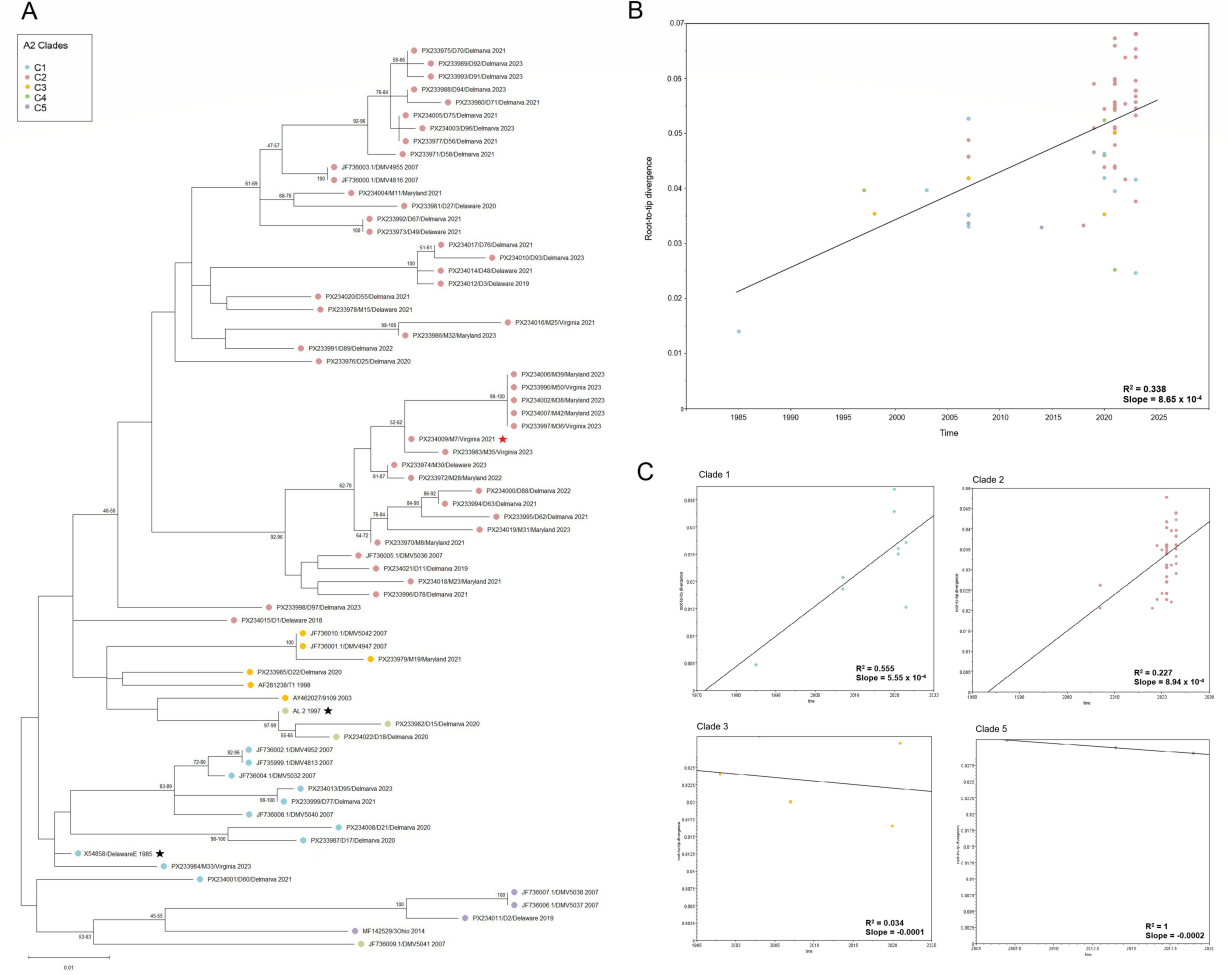
Phylogenetic trees and the corresponding root-to-tip divergence plots based on the HVR of the VP2 gene, focusing on US genogroup A2 strains. (**A**) The phylogeny was inferred using the maximum likelihood method and Tamura-Nei (1993) model, and the tree with the highest log likelihood is shown. The initial tree for the heuristic search was selected by choosing the tree with the superior log-likelihood between a Neighbor-Joining (NJ) tree and a Maximum Parsimony (MP) tree. The NJ tree was generated using a matrix of pairwise distances computed using the Tamura-Nei model. Evolutionary analyses were conducted and visualized in MEGA12. The tree was constructed using 53 HVR nucleotide sequences obtained from Sanger sequencing of the PCR products (GenBank accession numbers PX233970–PX234022) and the nucleotide sequences of 17 reference IBDV genogroup A2 US variant strains in GenBank (indicated by the remaining accession numbers). All the sequences were trimmed to nucleotides 649 to 1,125 of the VP2 gene, to be the same length. The tree was divided into the previously described Genogroup A2 five clades, each depicted in a different color. The reference strains Delaware E and AL-2 commonly used as vaccines are represented by a black star, and the strain that was further characterized in this study (M7/Virginia/2021) was represented by a red star. (**B**) Root-to-tip divergence plots of the VP2 HVR Genogroup A2 clades. The x-axis of the root-to-tip divergence plot shows the collection year, and the y-axis shows the root-to-tip divergence on the maximum-likelihood phylogenetic tree. The linear regression is represented by the black line of the root-to-tip divergence and collection year. Each IBDV strain is represented by a colored circle, in which the color represents the Genogroup A2 clade. (**C**) Individual Genogroup A2 clades root-to-tip divergence plots of the VP2 HVR. All the strains with four or more sequences available were analyzed.

The sequences of the HVR of VP2 (encoded by segment A) were obtained from 53 samples (GenBank accession numbers PX233970–PX234007 [partial VP2 sequences] and PX234008–PX234022 [whole VP2 sequences]), and the sequences of a 722-bp region of the VP1 Gene (encoded by segment B) was obtained from 34 samples (GenBank accession numbers PX234023–PX234051 [partial VP1 sequences] and PX233332–PX233336 [whole VP1 sequences]). The sequences of both the HVR and the VP1 gene were obtained from 28 samples (Table 1; Table S1). The sequences were used to construct phylogenetic trees for both the HVR and VP1 (Fig. 1). Phylogenetic analysis revealed that all the HVR sequences clustered in genogroup A2 (Fig. 1A, turquoise) and all the VP1 sequences clustered in genogroup B1 (Fig. 1B, blue). Of the A2 genogroup, the same five clades identified in samples from 2007 were present in the samples from 2018 to 2023. Clade 1 was found in 6/53 (11.3%) of the sequences, whereas clade 2 was found in 42 (79.2%), clade 3 in 2 (3.8%), clade 4 in 2 (3.8%), and clade 5 in 1 (1.9%) of the samples (Fig. 2A). Within the clade 2 viruses, there had been evolution into sub-clades. When considered together, root-to-tip analysis revealed there was not a strong linear clock-like evolution of the A2 viruses in Delmarva (Fig. 2B); however, when divided into individual clades and sub-clades, clade 1 and some sub-clades of clade 2 showed evidence of evolving in a linear clock-like fashion, whereas others did not (Fig. 2C; Fig. S1), suggesting the clades are evolving independently in parallel. Taken together, these data demonstrate that all the IBDV Delmarva strains for which we had both segment A and B sequences belonged to genogroup A2B1, which is consistent with them being US antigenic variant strains. Moreover, antigenic drift had occurred, further expanding the genetic diversity, and while the five clades persisted in the population, our data suggest they are evolving independently.

### Antigenic drift was observed in the VP2 capsid and an amino acid signature, “NRED,” was identified in the majority of sequences

**Fig 3:**
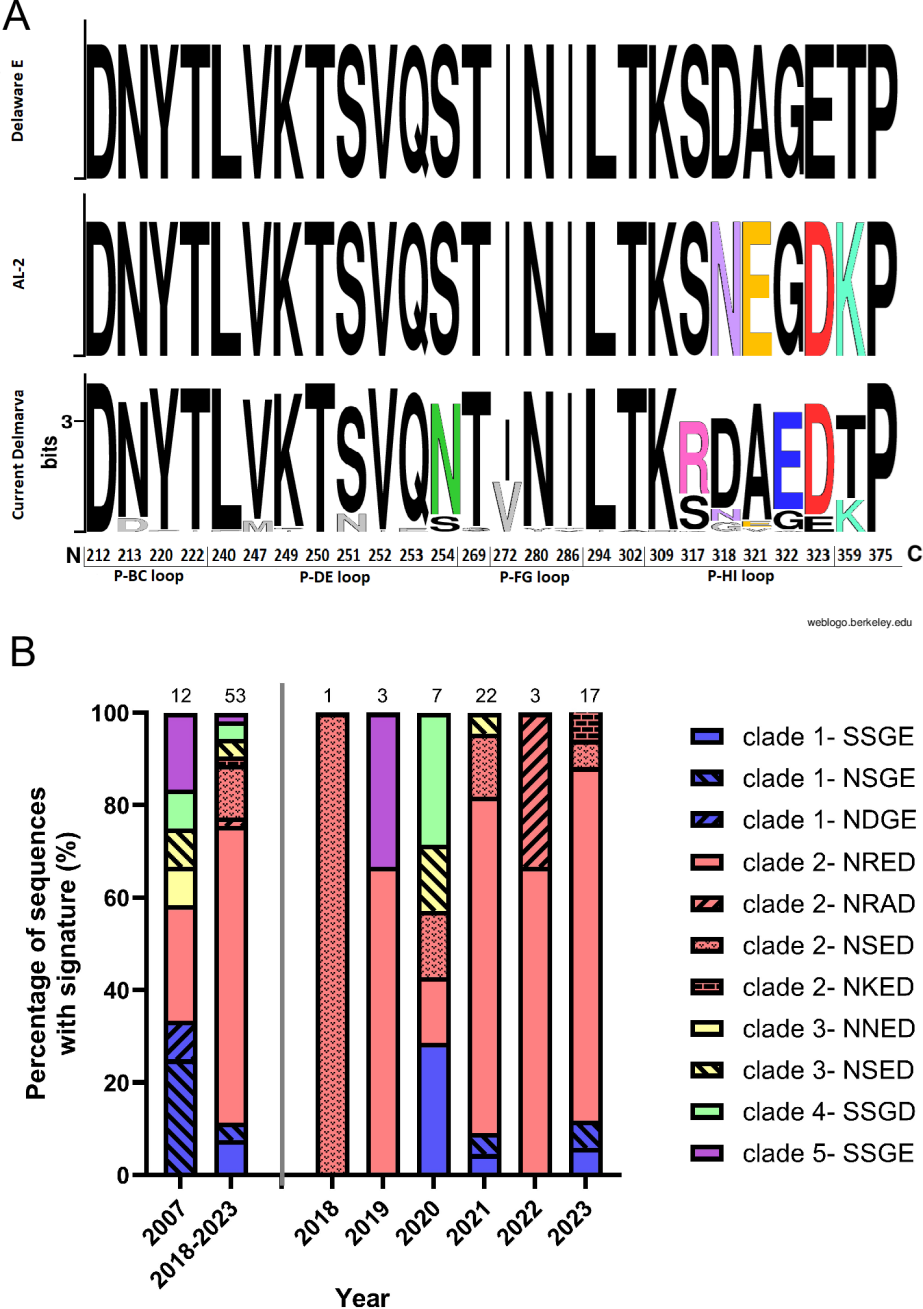
HVR sequence analysis. (**A**) A sequence logo plot of the HVR sequence was constructed using the alignment of the amino acid sequences of the 53 Delmarva region strains obtained between 2018 and 2023 (“Current Delmarva” panel) compared with two representative US variant genogroup A2 strains, Delaware E (upper panel) and AL-2 (middle panel). Each amino acid was depicted by its letter abbreviation. The black letters represent amino acids present in Delaware E, the colored letters represent amino acids that were not present in Delaware E. The numbers on the x-axis correspond to the amino acid position in the HVR, and the y-axis corresponds to the information content (bits). A fully conserved amino acid region has log_2_ (20) = 4.3 bits. (**B**) A stacked bar plot of the clades and amino acid signatures at amino acid residues 215, 317, 322, and 323. The y-axis depicts the percentage of the amino acid sequences that possessed a given signature, as defined in the legend, and the x-axis denotes the years of sampling. The numbers on top of each stacked bar represent the number of sequences.

When compared with the Del-E and AL-2 variants, 52/53 (98.1%) of the IBDV HVR sequences obtained in Delmarva from 2018 to 2023 contained amino acid substitutions, and of the 132 amino acids in the HVR, substitutions were identified at 24 (18.2%) sites (Fig. S2). The consensus sequence of the field strains contained four amino acid substitutions compared to Del-E: S254N, S317R, G322E, and E323D (“NRED”) (Fig. 3A; Fig. S2). These were observed in the second (P-DE) and fourth (P-HI) hydrophilic loops (Fig. S2), thought to be major sites of antibody binding, and were therefore likely to be the result of antigenic drift. The asparagine (N) at position 254 was observed in 46 (86.7%) of the sequences, the arginine (R) at position 317 was present in 35 (66%) of the sequences, the glutamic acid (E) was present in 43 (81.1%) of the sequences, and the aspartic acid (D) was present in 46 (86.8%) of the sequences. All four amino acid substitutions were present in 34 (64%) of the sequences. The E323D substitution was also found in the AL-2 strain, but the S254N, S317R, and G322E substitutions were not present in either Del-E or AL-2 (Fig. 3A). Previously, strains with this signature isolated in 2007 had been classified as clade 2 Del-E variants (20).

### Few amino acid substitutions were observed outside of the HVR

To determine whether amino acid substitutions occurred elsewhere in the capsid, the whole VP2 capsid gene was sequenced from 15 of the samples by MinION sequencing (GenBank accession numbers PX234008–PX234022) (Fig. S3). Of these sequences, all 15 contained amino acid substitutions within the HVR, but only 7 (46.6%) contained any substitutions outside of the HVR. Moreover, while substitutions were identified at 20 sites in the HVR, only seven sites outside of the HVR had substitutions, demonstrating that there were fewer amino acid substitutions outside of the HVR.

### The “NRED” HVR amino acid signature increased in prevalence over time and was present in 76% of the sequences in 2023

When the consensus sequence S254N, S317R, G322E, and E323D amino acid substitutions were quantified by year, all four substitutions (“NRED”) were found in 2/3 (67%) of the HVR sequences in 2019, 1/7 (14%) in 2020, 16/22 (72%) in 2021, 2/3 (67%) in 2022, and 13/17 (76%) in 2023 (Fig. 3B). Previously, clade 2 variant strains with this signature were found in 3/12 (25%) of the samples in 2007 (20). Taken together, the prevalence of clade 2 variant strains containing the “NRED” amino acid signature had increased from 25% in 2007 to 76% in 2023. Other signatures were also found in the samples, for example, compared with the Del-E “SSGE” signature, we detected viruses with one amino acid substitution (SSGD and NSGE), two substitutions (NDGE), three substitutions (NSED), and four substitutions (NKED, NNED, NRAD); however, the same increase in prevalence was not observed with the other signatures (Fig. 3B).

### AlphaFold 3 modeling revealed that some HVR amino acid substitutions face a canyon on the virus capsid surface

**Fig 4:**
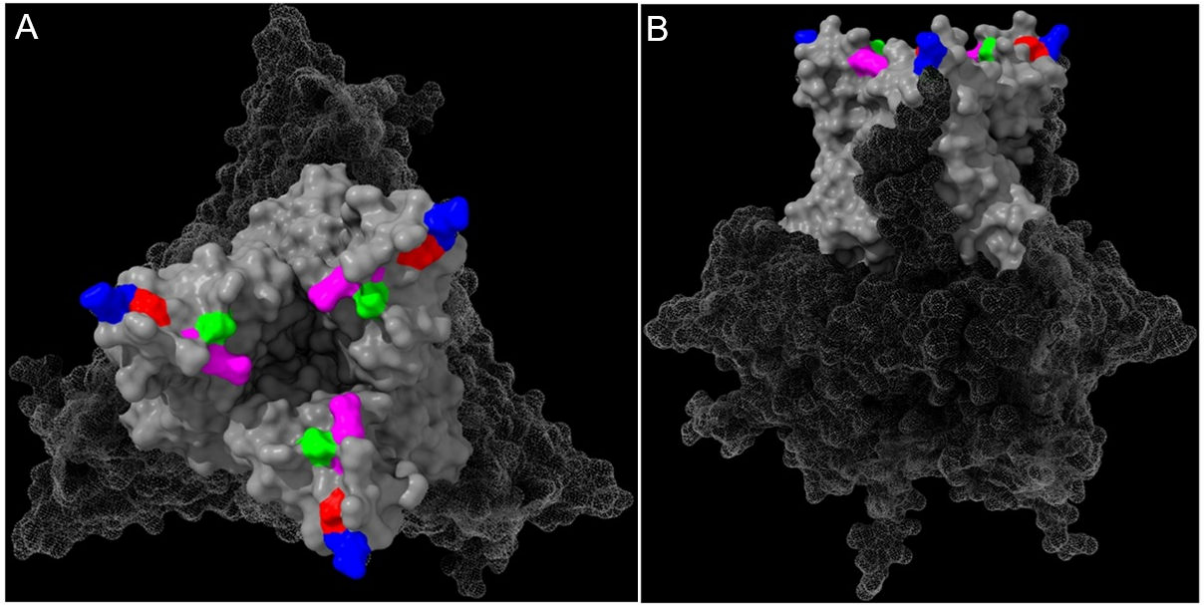
Structural model of the “NRED” genetic signature the predicted structure of the HVR Del-E trimer was modeled using the AlphaFold 3 multimer model. The predicted structure was loaded and visualized with PyMol, and the end-on/axial view (**A**) and side view (**B**) were displayed. The Del-E atoms were depicted in gray, with the HVR solid and the rest of the VP2 trimer meshed. The positions of the amino acid substitutions were mapped onto the structure and highlighted (S254N, green; S317R, purple; G322E, blue; E323D, red).

AlphaFold 3 was used to model the VP2 homotrimer, and the consensus level amino acid substitutions were highlighted (Fig. 4). Amino acid substitutions S254N and S317R, although 61 residues apart in the HVR sequence, were predicted to be adjacent to each other, on the face of the VP2 HVR oriented towards a canyon into which the receptor could potentially bind (Fig. 4), suggesting that the identity of the amino acid residues at these sites could influence receptor binding, or the binding of antibodies that sterically hinder virus-receptor interactions.

### The “NRED” HVR amino acid signature was associated with a reduced virus neutralization (VN) antibody titer

**Fig 5:**
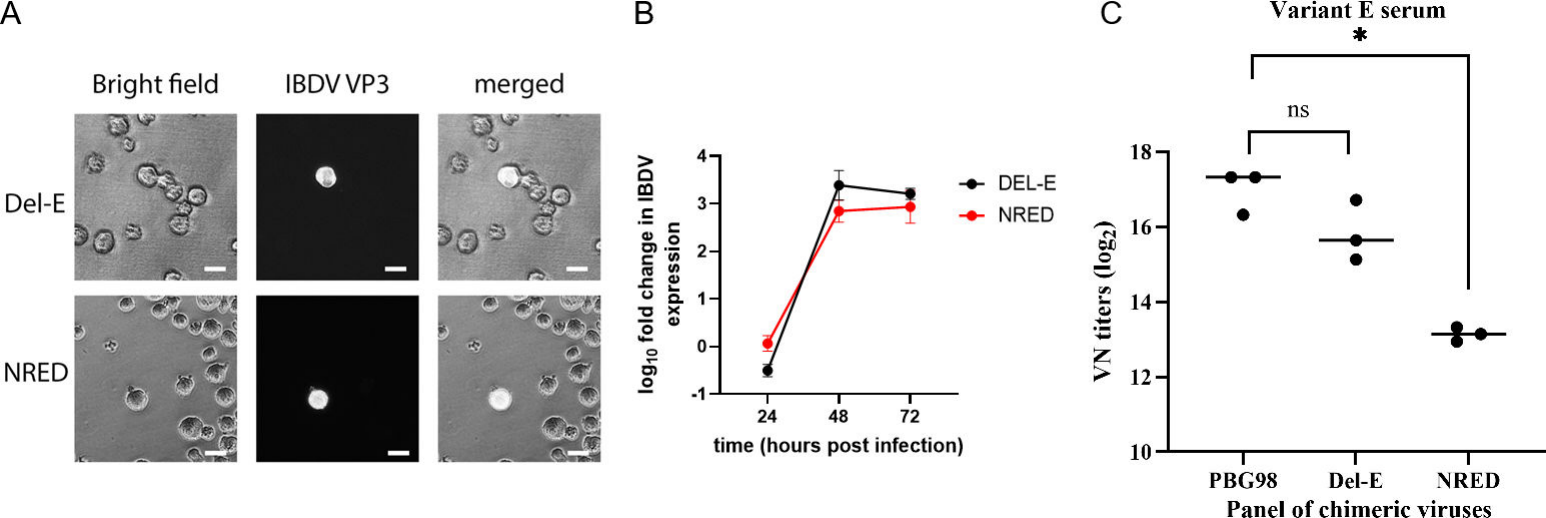
Rescue, replication, and neutralization of PBG98-HVR^DEL-E^ and PBG98-HVR^NRED^ IBDV strains in DT40 cells. (**A**) Immunofluorescence microscopy of DT40 cells following virus rescue, showing viral protein VP3 expression. Cells infected with PBG98-HVR^DEL-E^ (Del-E) and PBG98-HVR^NRED^ (NRED) were imaged under bright-field and VP3-specific immunofluorescence to confirm successful viral rescue (scale bars, 20 µm). (**B**) Replication kinetics of PBG98-HVR^DEL-E^ and PBG98-HVR^NRED^ IBDV strains in DT40 cells, expressed as log₁₀ fold change in IBDV genome expression, normalized to a housekeeping gene (RPLPO) and expressed relative to mock-infected cells in a ΔΔCt analysis. Data points represent mean values, with error bars indicating standard deviation. (**C**) A virus neutralization assay was performed in triplicate in DT40 cells using commercially available hyperimmune serum from chickens immunized with the Del-E variant, and the end-point determined by immunofluorescence microscopy using a mouse-anti-IBDV VP3. The highest dilution of serum where there were no IBDV-antigen positive cells was considered as the virus neutralization (VN) titer, which was expressed as log2. The neutralization titer of PBG98-HVR^DEL-E^ and PBG98-HVR^NRED^ was compared with the PBG98 control. *P < 0.05; ns, not significant.

Using reverse genetics, chimeric recombinant molecular clones of IBDV were generated in the backbone of the laboratory-adapted strain PBG98 that contained the HVR from either the Del-E strain (PBG98-HVR^DEL-E^) or a Del-E variant containing the S254N, S317R, G322E, and E323D amino acid substitutions (PBG98-HVR^NRED^). The plasmids were electroporated into the immortalized chicken B-cell line, DT40, to rescue the viruses, as previously described (4, 36), and rescue was confirmed by immunofluorescence microscopy (IFM) using an in-house antibody raised against VP3 (Fig. 5A). Viruses were passaged up to five times in DT40 cells, and the replication kinetics of the viruses were then compared by RT-qPCR (Fig. 5B). Briefly, DT40 cells were infected at a multiplicity of infection (MOI) of 0.01, and the viral genome copy number quantified at 24, 48, and 72 h post-infection (hpi). The PBG98-HVR^NRED^ virus was found to replicate with similar kinetics to the PBG98-HVR^DEL-E^ virus but had a lower peak titer (Fig. 5B). Hyperimmune serum (var-E) was obtained from AVSBio (formerly Charles River) that had been generated by serially inoculating chickens with Del-E, and a microneutralization assay was performed in triplicate using DT40 cells to evaluate the VN antibody titer. The VN titer was reduced from a mean of 16.99 (standard deviation, SD, 0.58) against PBG98 and 15.83 (SD, 0.80) against the PBG98-HVR^DEL-E^ virus to a mean of 13.14 (SD, 0.18) against the PBG98-HVR^NRED^ virus (*P* < 0.05) (Fig. 5C), demonstrating that the “NRED” amino acid signature significantly reduced neutralizing antibody responses.

### Amino acid substitutions were observed in the VP1 polymerase encoded by segment B

Among the 34 partial VP1 sequences obtained from 2018 to 2023 in Delmarva, 20 (58%) contained amino acid substitutions, at a total of 14 sites compared with Del-E (Fig. S4). Moreover, 16/34 (47%) contained substitutions within a triplet of amino acids at positions 145, 146, and 147, which have previously been implicated in IBDV virulence (10, 18, 37). To determine whether amino acid substitutions occurred elsewhere in the polymerase, the whole VP1 gene was sequenced from five of the samples by MinION sequencing (GenBank accession numbers PX233332–PX233336), and a genetic signature of five amino acid substitutions (compared with Del-E) was identified in 4/5 (80%) of the sequences: I385V, R508K, K579R, R682K, and S718N (Fig. S5). These amino acid substitutions were mapped onto the VP1 predicted structure for strain Del-E (Fig. S6). Briefly, the VP1 protein showed a centrally located catalytic cleft, consistent with known RNA-dependent RNA polymerase architecture. The amino acid triplet at positions 145–147 formed a patch adjacent, but not within, this cleft (Fig. S6, orange highlighted region), and the five amino acid substitutions I385V, R508K, K579R, R682K, and S718N were distributed over the surface of the protein (Fig. S6, blue highlighted regions).

## DISCUSSION

The last molecular characterization of IBDV strains circulating in the Delmarva region isolated strains in 2007. In the present study, we aimed to provide more up-to-date information on the strains circulating in the region. To this end, the sequences of the HVR of the VP2 gene encoded by Segment A, and a region of the VP1 polymerase gene encoded by Segment B, were obtained from 53 and 34 samples, respectively, from bursal samples obtained from 2018 to 2023. Phylogenetic analysis revealed that all sequences belonged to genogroup A2 and B1 (Fig. 1), which is typical of US variant strains. Importantly, we did not find any evidence of exotic strains in our samples, despite the recent spread of nv/distinct A2dB1b strains from Asia to Africa and S. America, the high prevalence of vv A3B2 strains in many countries, and the rise of reassortant A3B1 strains in Europe.

In 2007, five clades of IBDV derived from the parental Del-E strain were reported to be circulating in Delmarva. At the time, clade 1 was found in 4/12 (33%) sequences (DMV4813, DMV4952, DMV5040, and DMV5032), clade 2 was found in 3/12 (25%) sequences (DMV4816, DMV4955, and DMV5036), clade 3 was found in 2/12 (17%) of the sequences (DMV4947 and DMV5042), clade 4 was found in 1/12 (8%) of the sequences (DMV5041), and clade 5 was found in 2/12 (17%) of the samples (DMV5037 and DMV5038) (20). In the present study, phylogenetic analysis revealed that the five clades continued to circulate in Delmarva, but the prevalence of each differed compared with 2007, with clade 2 viruses being substantially overrepresented and the other clades underrepresented in the sequences (Fig. 2): Clade 1 was found in 6/53 (11.3%) of the sequences, clade 2 was found in 42/53 (79.2%) samples, clade 3 was found in 2/53 (3.8%) of the samples, clade 4 was found in 2/53 (3.8%) of the samples, and clade 5 was found in 1/53 (1.9%) of the samples. Therefore, while the clade 2 viruses were only in 25% of the sequences in 2007, they were found in 79% of the sequences in 2018–2023. Moreover, we determined that within the clade 2 viruses, there had been evolution into additional sub-clades, demonstrating this clade has increased in diversity over the last 18 years. Root-to-tip analysis of a temporal phylogenetic tree revealed that clade 1 and some sub-clades of clade 2 showed evidence of evolving in a linear clock-like fashion, whereas others did not (Fig. 2; Fig. S1), suggesting the clades are evolving independently in parallel. This independent evolutionary pattern may imply that IBDV evolutionary dynamics need to be analyzed at the clade level, or sub-clade level.

Alignments of the HVR sequences in the samples obtained in 2007 revealed that 34 (86%) of the clade 2 viruses contained the amino acid substitutions S254N, S317R, G322E, and E323D compared with the Del-E strain. This “NRED” amino acid signature has therefore increased in prevalence in Delmarva from 25% in 2007 to 64% in 2018–2023. Furthermore, we found this signature was present in 76% of the samples in 2023 (Fig. 3B). Other signatures were also found in the samples, for example, compared to the Del-E “SSGE” signature, we detected viruses with one amino acid substitution (SSGD and NSGE), two substitutions (NDGE), three substitutions (NSED), and four substitutions (NKED, NNED, NRAD) (Fig. 3B); however, the same increase in prevalence seen with the NRED signature was not observed with the other signatures.

It remains unknown why the “NRED” amino acid signature has increased in prevalence over time, but we hypothesized that it could provide a fitness advantage to the IBDV population over the parental Del-E strain, or other Delmarva clades. Consistent with this hypothesis, we discovered that the amino acid signature was responsible for an 8-fold reduction in VNT of hyperimmune serum raised against the Del-E strain, from approximately 15 log_2_ to 13 log_2_ that was statistically significant (*P*<0.05) (Fig. 5C), suggesting that it could drive immune escape. It is important to note that the VN assay was performed using hyperimmune serum and hence the high titers, and it will be important to evaluate the cross-neutralization in chickens with more biologically relevant antibody titers in the future. One advantage of our approach is that we were able to engineer chimeric IBDV viruses by reverse genetics in the background of our lab-adapted strain, PBG98, thus isolating the effect of the signature from any other mutations that may have been present, and confirming that the S254N, S317R, G322E, and E323D amino acid substitutions alone contributed to the reduction in antibody neutralization. Using this same approach, we have previously reported an eightfold reduction in the VNT (from approximately 15 log_2_ to 13 log_2_) when hyperimmune serum raised against a genogroup A1 strain (F5/70) was used to neutralize a chimeric strain with a vv genogroup A3 HVR (UK661) (36). Despite this, genogroup A1 vaccines are still used to provide clinical protection against disease caused by infection with genogroup A3 strains in the field. Therefore, we hypothesize that vaccines based on Del-E will still provide some cross-protective immunity against the NRED challenge in the field. However, this will need to be experimentally tested *in vivo*. Furthermore, it may seem counterintuitive that the serum in this study neutralized the PBG98 control virus (genogroup A1) as well as Del-E; however, it is known that genogroup A2 vaccines can provide cross-protection against an A1 challenge (38), and these data are consistent with that finding.

Next, we evaluated the replication kinetics of the chimeric viruses in the chicken B cell line DT40. The PBG98-HVR^NRED^ virus did not replicate significantly faster, or to significantly higher titers than the PBG98-HVR^DEL-E^ virus, suggesting that the reduced neutralization titer we observed was not the result of increased viral fitness, but of altered antigenicity. Structural modeling revealed that although S254N and S317R were separated from each other in sequence, they were adjacent to each other on the predicted VP2 structure (Fig. 4, green and purple residues). Moreover, the VP2 protein forms a trimer as it assembles into the capsid, and by modeling the trimer, we demonstrated that the S254N and S317R substitutions faced a canyon on the virus surface. The precise mechanism of how the IBDV capsid interacts with candidate cellular receptors remains unknown, but it is possible that receptors bind within the canyon, as is the case for some members of the *Picornaviridae* family (39). If this is the case, then antibodies that bind epitopes that include 254S and/or 317S could sterically inhibit receptor binding. Substituting these sites to 254N or 317R could circumvent antibody binding, thus reducing their ability to neutralize infection. Moreover, arginine (R) is positively charged, meaning that S317R substitution could change the charge in the canyon, potentially influencing receptor or antibody binding. Although G322E and E323D do not face the canyon directly, amino acid substitutions at these sites could lead to conformational changes elsewhere in the trimer. This work highlights the importance of conducting structural modeling and not relying on sequence data alone.

In the VP1 polymerase gene, a triplet of amino acids at positions 145, 146, and 147 is associated with IBDV virulence (37). Non-vv strains typically have amino acids asparagine (N), glutamic acid (E), and glycine (G) at these sites, forming a “NEG” triplet, although non-vv strains have also been identified in the US with aspartic acid (D) or serine (S) at position 147 (forming a “NED” or “NES” triplet), and a threonine (T) or serine (S) at position 145, forming a “TEG” or “SEG” triplet (20). In contrast, vv viruses with a threonine, aspartic acid, and asparagine or serine (TDN or TDS) triplet have an increased virulence (37). In the present study, we observed that although the Segment B sequences all belonged to genogroup B1, 18/34 (53%) of our samples contained an NED triplet, whereas 7/34 (21%) had NEG, 3/34 (9%) SED, 2/34 (6%) DEG, 1/34 (3%) SEG, 1/34 (3%) GEG, 1 (3%) NET, and 1 (3%) NDS (Fig. S4). There was therefore considerable heterogeneity at these sites, more than previously described, and it will be beneficial to evaluate if there are differences in the virulence of strains with differing triplet sequences, particularly the strain containing NDS, as it only differs from the TDS triplet associated with increased virulence by one amino acid. It remains unknown why this triplet is associated with virulence, but the VP1 gene encodes the polymerase, which replicates the genome, so modifications to the triplet could potentially influence viral genome replication kinetics. VP1 is also known to be present within cytoplasmic virus factories (VFs), where it interacts with other viral proteins, such as VP3 (40, 41), and it is post-translationally modified by host-cell proteins (42–44). Although the triplet has not been implicated directly in known VP1 interactions within the cell, we discovered that it is predicted to form a patch on the surface of the molecule (Fig. S6), and it is therefore possible that as yet undefined interactions are mediated via the triplet, which may in turn influence viral replication and virulence; however, this also remains to be tested. In addition, five amino acid substitutions (I385V, R508K, K579R, R682K, and S718N) were observed in 4/5 (80%) of the full-length VP1 sequences, and it is also possible that these substitutions cause conformational changes in the VP1 that alter the kinetics of genome replication or interactions with cellular or viral proteins, which should also be tested experimentally.

Our study is not without limitations. For example, it remains to be determined whether the reduction in VN titer we observed due to the HVR “NRED” signature manifests in an increased likelihood of vaccine failure in the field. To determine this, an *in vivo* challenge study would be necessary. In a study published by Gelb et al. (20), clade 5 viruses demonstrated an enhanced ability to break through vaccine-induced protective immunity when a Del-E vaccine was used (20). However, the authors did not report conducting a challenge study with the other clades. Given the high prevalence of clade two viruses in Delmarva in recent years, it would be beneficial to determine if these viruses escape vaccine-induced protection compared to other clades. We also did not perform whole genome sequencing (WGS) on the majority of our samples, and so genetic analysis was largely limited to Sanger sequences of the HVR of the VP2 gene or a 722 bp region of the VP1 gene. However, we obtained sequences of the whole VP2 capsid gene from 15 samples, and the whole VP1 polymerase gene from five samples, by MinION sequencing. Using this approach, we discovered that few amino acid substitutions were present elsewhere in the VP2, and the majority of changes were in the VP2 HVR, confirming that our phylogenetic analyses were meaningful. Recently, WGS has been applied to IBDV strains using next-generation sequencing approaches (45, 46). It would be beneficial to apply this technology to determine the sequences of the whole genomes of IBDV field strains in the future. Finally, our analyses were limited to samples obtained in Delaware, Maryland, and Virginia, and it will be beneficial to determine the prevalence of the clade 2 Del-E variants bearing the “NRED” signature in other states, to determine if this is a local/regional phenomenon, or whether this clade is now dominant within the US nation-wide.

In summary, all IBDV sequences in Delmarva 2018–2023 belonged to the A2 and B1 genogroups, typical of US variant strains. Multiple clades were found to be circulating, and clade 2 viruses, originally described in 25% of the samples in 2007, now predominated and were present in 76% of the samples in 2023. Viruses in this clade contained HVR amino acid substitutions S254N, S317R, G322E, and E323D, which significantly reduced the VN titer (*P* < 0.05), and we hypothesize this amino acid signature increased biological fitness of the strain in the population by driving immune escape. Heterogeneity was observed in the VP1 sequences in and around an amino acid triplet at residues 145–147, which is known to influence virulence. The consequences of the HVR and VP1 mutations in terms of pathogenesis, clinical presentation, immunosuppressive potential, and vaccine failure remain poorly understood, and further studies are required to improve IBDV disease control.

## SUPPLEMENTAL MATERIAL

Fig. S1 spectrum.02976-25-s0001.tif Phylogenetic tree and the corresponding root-to-tip divergence plots based on the HVR of the VP2 gene, focusing on US genogroup A2 clade 2 sub-clades

Fig. S2 spectrum.02976-25-s0002.tif Alignment of the VP2 HVR amino acid sequences from Delmarva in 2018–2023 compared to the Delaware E and AL-2

Fig. S3 spectrum.02976-25-s0003.tif Alignment of the full-length VP2 amino acid sequences from Delmarva in 2018–2023 compared with the Del-E variant

Fig. S4 spectrum.02976-25-s0004.tif Alignment of the partial VP1 amino acid sequences from Delmarva in 2018–2023 compared with the Del-E variant

Fig. S5 spectrum.02976-25-s0005.tif Alignment of the full-length VP1 amino acid sequences from Delmarva compared to the Del-E variant

Fig. S6 spectrum.02976-25-s0006.tif Structural modeling of the VP1 protein

Table S1 spectrum.02976-25-s0007.pdf Metadata, clinical findings, diagnostic results, and sequencing information for bursal samples collected in the Delmarva region from 2018 to 2023

Supplemental figure legends spectrum.02976-25-s0008.docx Supplemental figure legends

ASM does not own the copyrights to Supplemental Material that may be linked to, or accessed through, an article. The authors have granted ASM a non-exclusive, world-wide license to publish the Supplemental Material files. Please contact the corresponding author directly for reuse.
