# Supplementary figures and images for: Molecular characterization of infectious bursal disease virus (IBDV) strains of genogroup A2B1 circulating in Delaware, Maryland, and Virginia from 2018 to 2023
Source: Microbiol Spectr. 2026 Apr 16;14(6):e02976-25. doi: 10.1128/spectrum.02976-25 (PMC13227996; doi:10.1128/spectrum.02976-25)

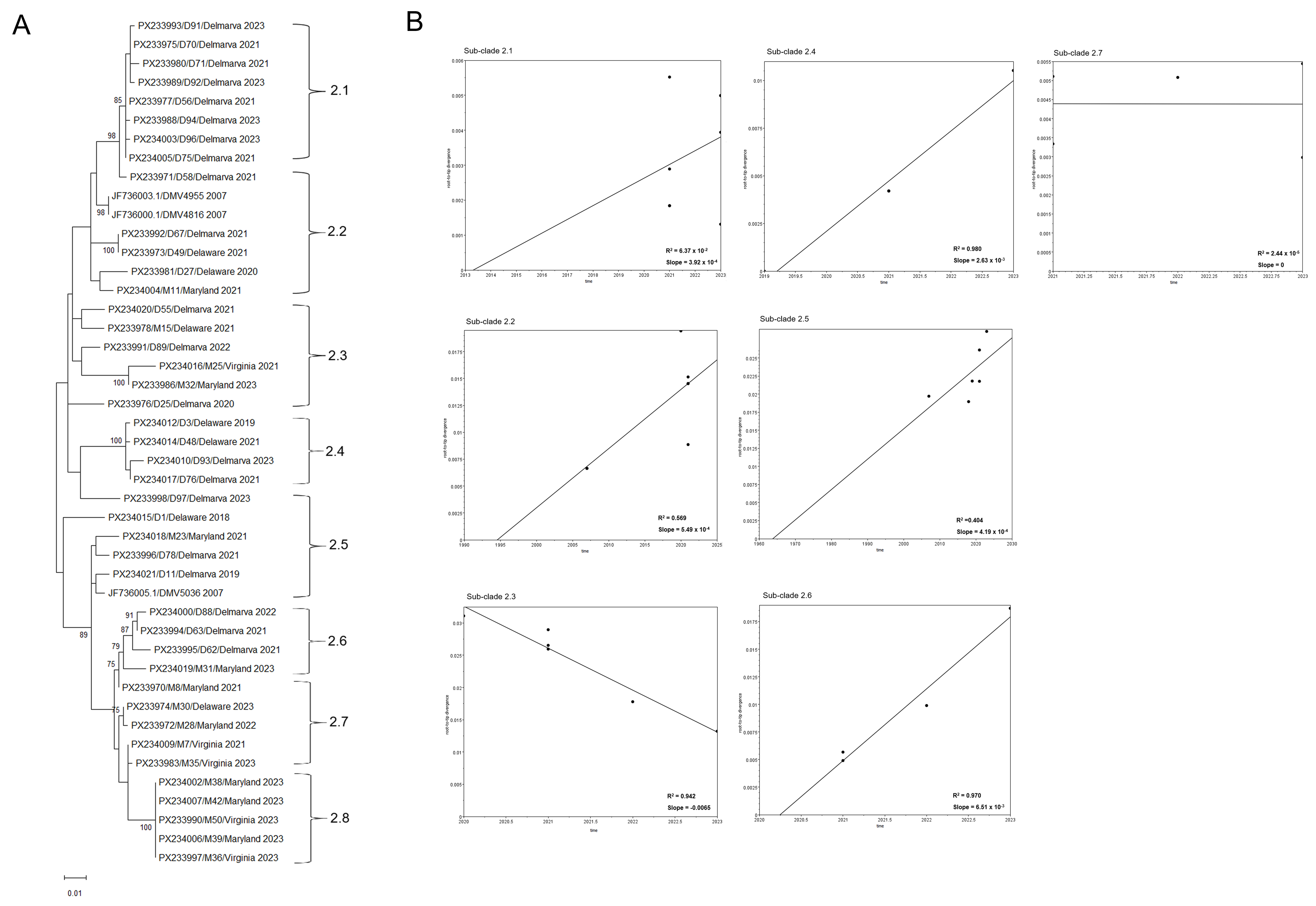

Supplement: Fig. S1 — Phylogenetic tree and the corresponding root-to-tip divergence plots based on the HVR of the VP2 gene, focusing on US genogroup A2 clade 2 sub-clades [file spectrum.02976-25-s0001.tif]

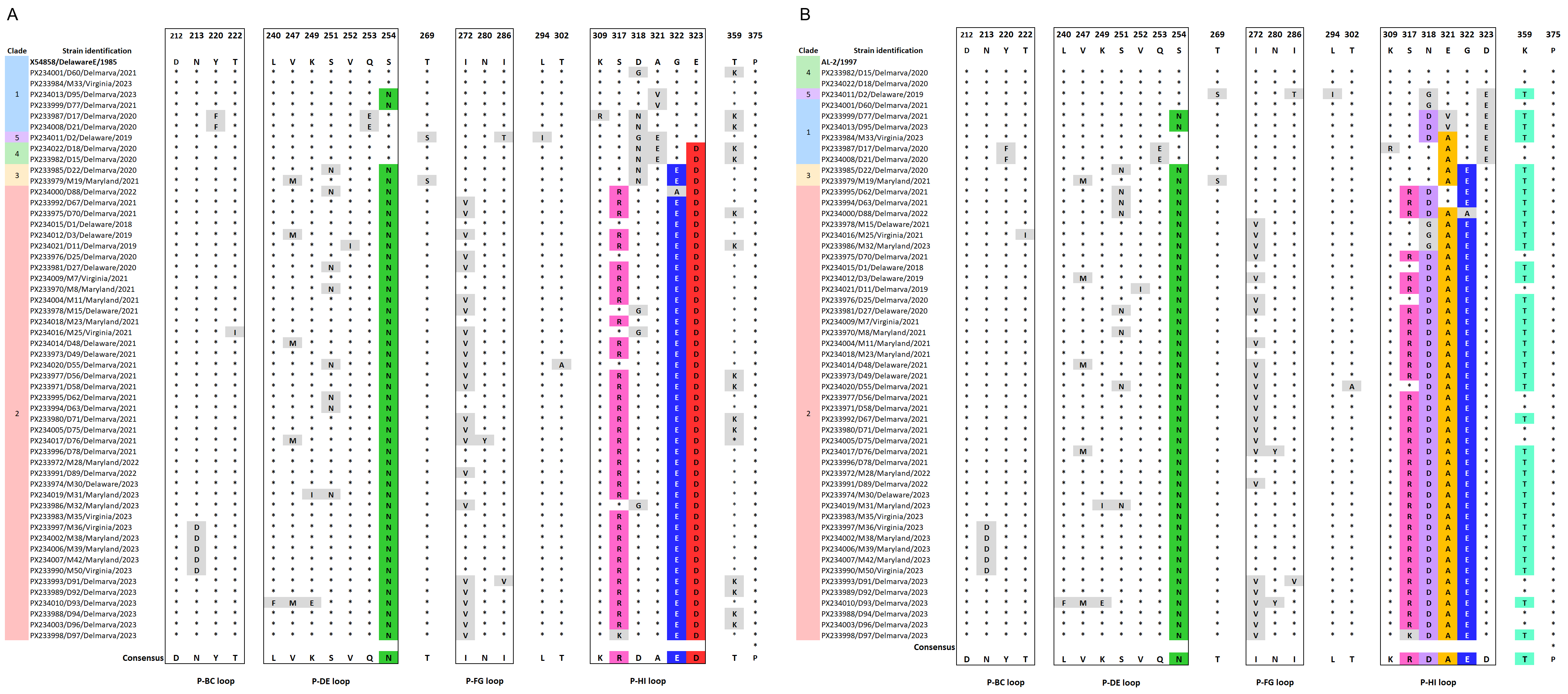

Supplement: Fig. S2 — Alignment of the VP2 HVR amino acid sequences from Delmarva in 2018–2023 compared to the Delaware E and AL-2 [file spectrum.02976-25-s0002.tif]

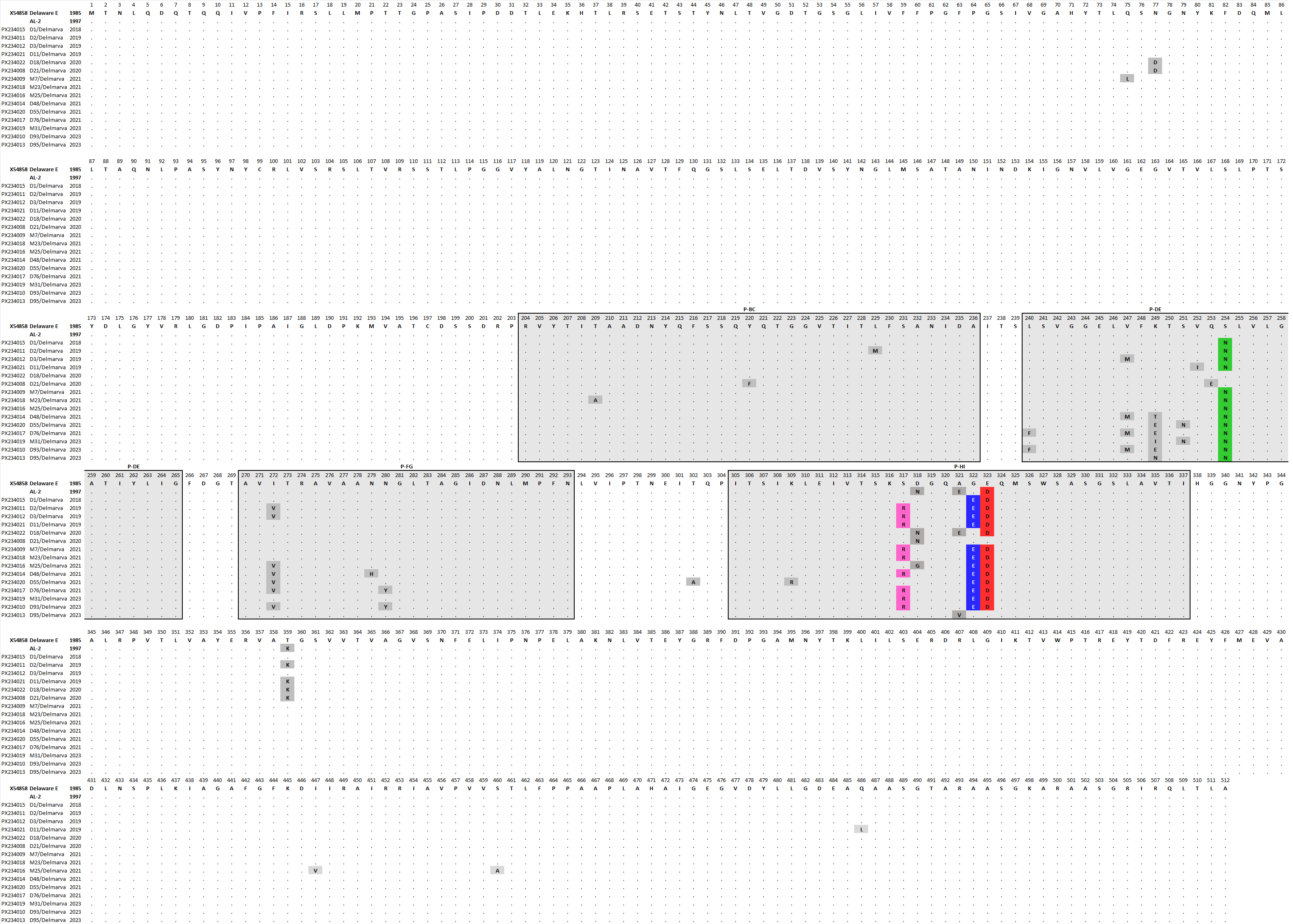

Supplement: Fig. S3 — Alignment of the full-length VP2 amino acid sequences from Delmarva in 2018–2023 compared with the Del-E variant [file spectrum.02976-25-s0003.tif]

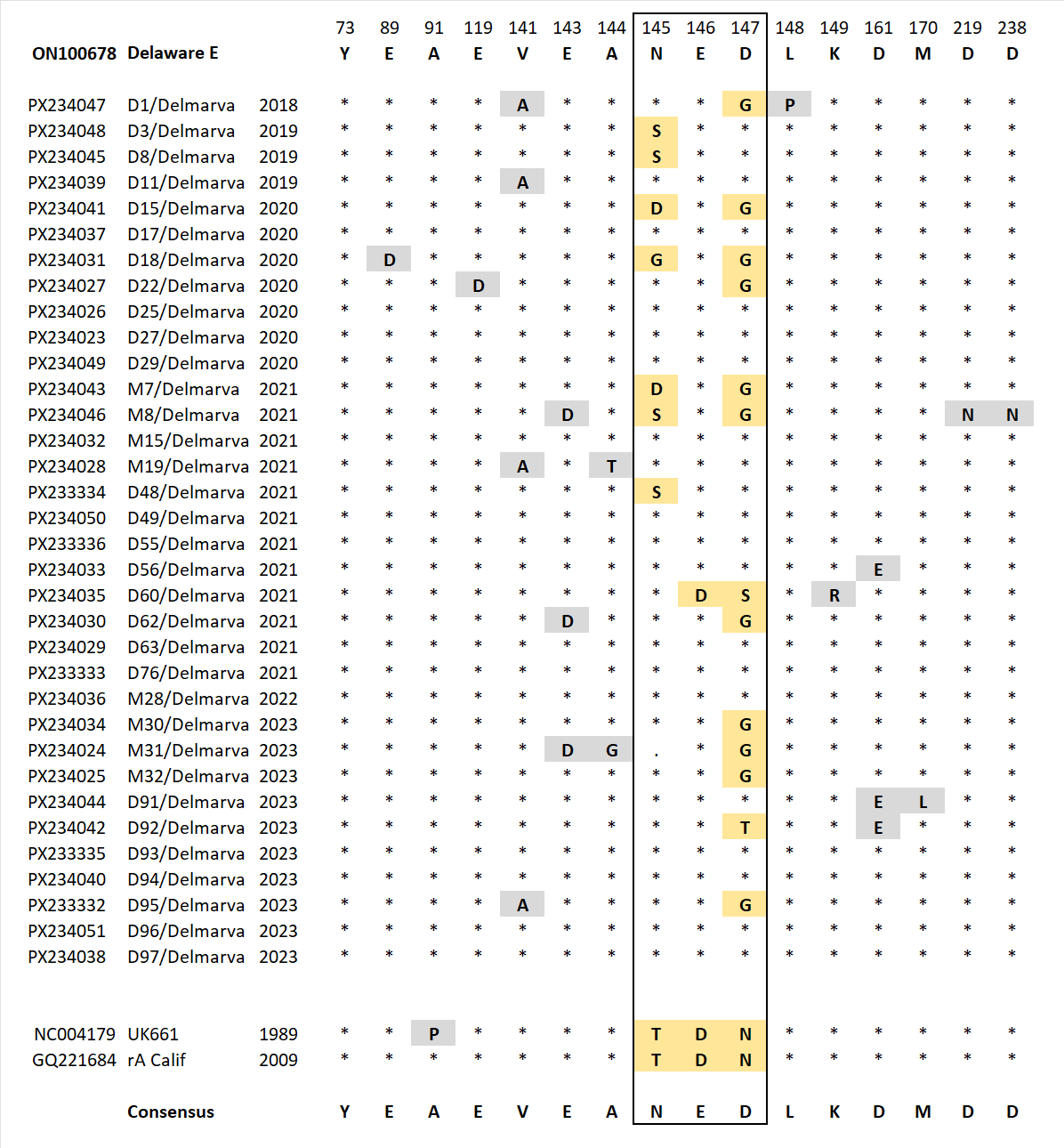

Supplement: Fig. S4 — Alignment of the partial VP1 amino acid sequences from Delmarva in 2018–2023 compared with the Del-E variant [file spectrum.02976-25-s0004.tif]

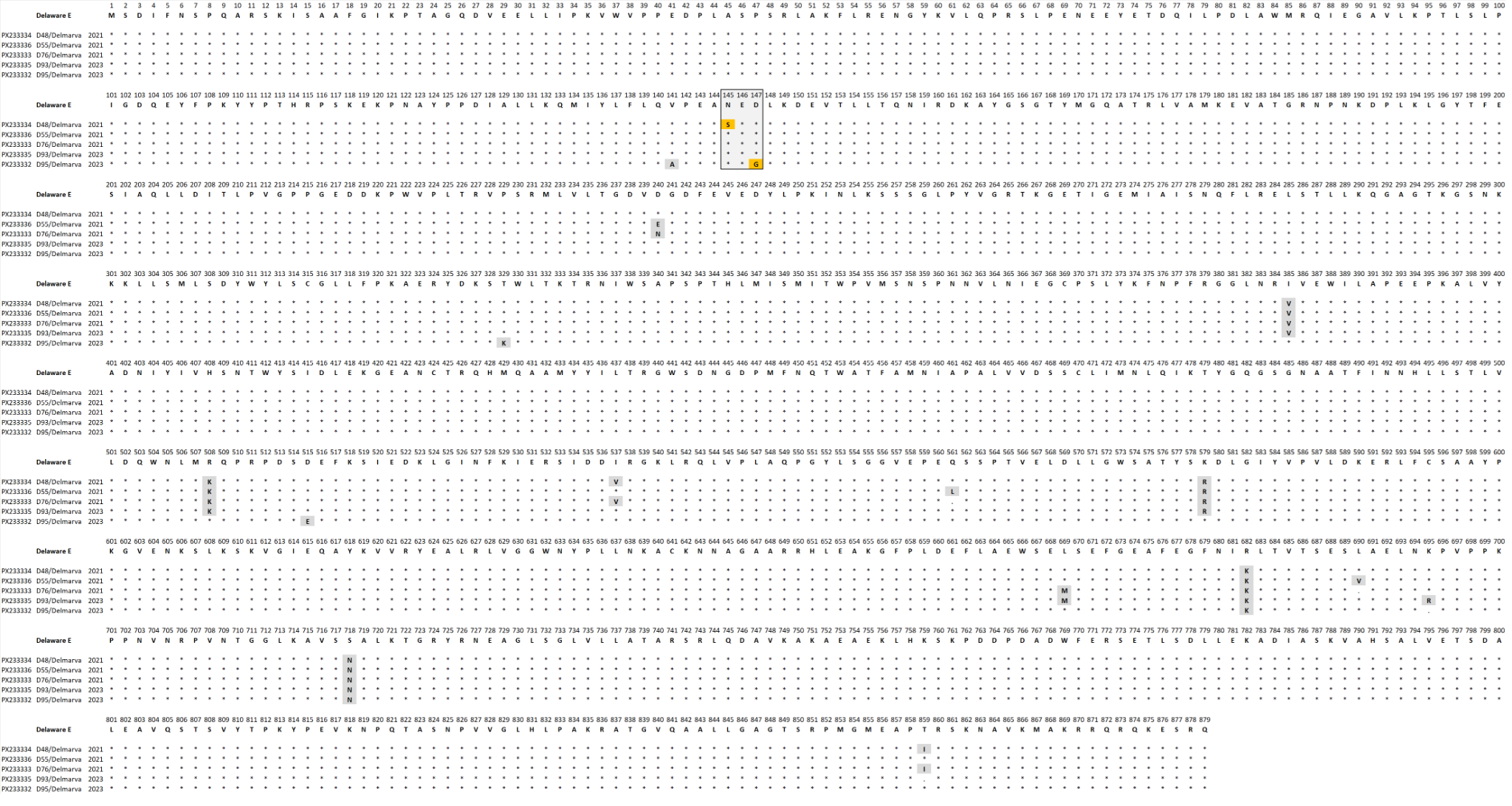

Supplement: Fig. S5 — Alignment of the full-length VP1 amino acid sequences from Delmarva compared to the Del-E variant [file spectrum.02976-25-s0005.tif]

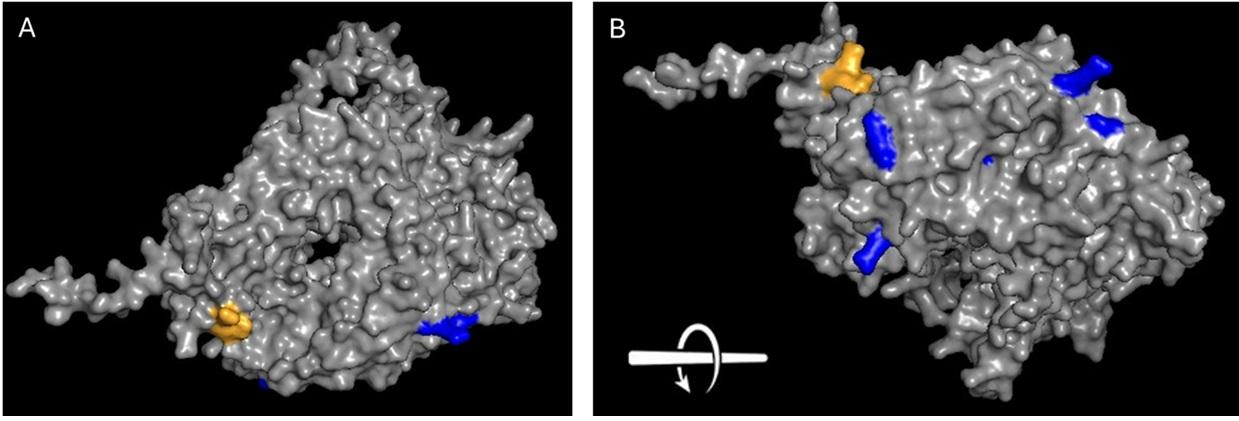

Supplement: Fig. S6 — Structural modeling of the VP1 protein [file spectrum.02976-25-s0006.tif]
