# Supplementary material for: Molecular characterization of infectious bursal disease virus (IBDV) strains of genogroup A2B1 circulating in Delaware, Maryland, and Virginia from 2018 to 2023
Source: Microbiol Spectr. 2026 Apr 16;14(6):e02976-25. doi: 10.1128/spectrum.02976-25 (PMC13227996; doi:10.1128/spectrum.02976-25)
Supplement: Table S1 — Metadata, clinical findings, diagnostic results, and sequencing information for bursal samples collected in the Delmarva region from 2018 to 2023 [file spectrum.02976-25-s0007.pdf]

Lab code	Submission date	Region	Birds type	Age (days)	Mortality (daily)	Bursa macroscopic lesions	Bursa microscopic lesions	Concomitant infections	IBDV RT-PCR (partial VP1 and/or HVR-VP2)	IBDV RT-PCR HVR-VP2	IBDV RT-PCR partial VP1	HVR-VP2 Sequence	Partial VP1 Sequence	RT-PCR Avian reovirus	RT-PCR Avian rotavirus	RT-PCR Chicken astrovirus	Genbank code HVR-VP2	Genbank code Partial-VP1	Genbank code Whole VP2	Genbank code Whole VP1
D1/Delmarva	2018	Delaware	Commercial	24	*	*	*	*	+	+	+	+	+	-	-	-	n/a	PX234047	PX234015	n/a
D2/Delmarva	2019	Delaware	Sentinel	*	*	*	*	*	+	+	-	+	-	-	-	-	n/a	n/a	PX234011	n/a
D3/Delmarva	2019	Delaware	Sentinel	*	*	*	*	*	+	+	+	+	+	-	-	-	n/a	PX234048	PX234012	n/a
D4/Delmarva	2019	Delaware	Sentinel	*	*	*	*	*	-	-	-	-	-	n/a	n/a	n/a	n/a	n/a	n/a	n/a
D5/Delmarva	2019	Delaware	Sentinel	*	*	*	*	*	+	+	-	-	-	-	-	-	n/a	n/a	n/a	n/a
D6/Delmarva	2019	Delaware	Sentinel	*	*	*	*	*	+	+	-	-	-	-	-	-	n/a	n/a	n/a	n/a
D7/Delmarva	2019	Delmarva	Sentinel	*	*	*	*	*	+	+	-	-	-	-	-	-	n/a	n/a	n/a	n/a
D8/Delmarva	2019	Delmarva	Sentinel	*	*	*	*	*	+	+	+	-	+	-	-	-	n/a	PX234045	n/a	n/a
D9/Delmarva	2019	Delmarva	Sentinel	*	*	*	*	*	+	+	-	-	-	-	-	-	n/a	n/a	n/a	n/a
D10/Delmarva	2019	Delmarva	Sentinel	*	*	*	*	*	+	+	-	-	-	-	-	-	n/a	n/a	n/a	n/a
D11/Delmarva	2019	Delmarva	Sentinel	*	*	*	*	*	+	+	+	+	+	+	-	-	n/a	PX234039	PX234021	n/a
D12/Delmarva	2019	Delmarva	Sentinel	*	*	*	*	*	+	+	-	-	-	+	-	-	n/a	n/a	n/a	n/a
D13/Delmarva	2020	Delmarva	Sentinel	*	*	*	*	*	-	-	-	-	-	n/a	n/a	n/a	n/a	n/a	n/a	n/a
D14/Delmarva	2020	Delmarva	Sentinel	*	*	*	*	*	+	+	-	-	-	-	-	-	n/a	n/a	n/a	n/a
D15/Delmarva	2020	Delmarva	Sentinel	*	*	*	*	*	+	+	+	+	+	-	-	-	PX233982	PX234041	n/a	n/a
D16/Delmarva	2020	Delmarva	Sentinel	*	*	*	*	*	-	-	-	-	-	n/a	n/a	n/a	n/a	n/a	n/a	n/a
D17/Delmarva	2020	Delmarva	Sentinel	*	*	*	*	*	+	+	+	+	+	-	-	-	PX233987	PX234037	n/a	n/a
D18/Delmarva	2020	Delmarva	Sentinel	*	*	*	*	*	+	+	+	+	+	-	-	-	n/a	PX234031	PX234022	n/a
D19/Delmarva	2020	Delmarva	Sentinel	*	*	*	*	*	+	+	-	-	-	-	-	-	n/a	n/a	n/a	n/a
D20/Delmarva	2020	Delmarva	Sentinel	*	*	*	*	*	+	+	-	-	-	-	-	-	n/a	n/a	n/a	n/a
D21/Delmarva	2020	Delmarva	Sentinel	*	*	*	*	*	+	+	-	+	-	-	-	-	n/a	n/a	PX234008	n/a
D22/Delmarva	2020	Delmarva	Sentinel	*	*	*	*	*	+	+	+	+	+	-	-	-	PX233985	PX234027	n/a	n/a
D23/Delmarva	2020	Delmarva	Sentinel	*	*	*	*	*	-	-	-	-	-	n/a	n/a	n/a	n/a	n/a	n/a	n/a
D24/Delmarva	2020	Delmarva	Sentinel	*	*	*	*	*	-	-	-	-	-	n/a	n/a	n/a	n/a	n/a	n/a	n/a
D25/Delmarva	2020	Delmarva	Sentinel	*	*	*	*	*	+	+	+	+	+	-	-	-	PX233976	PX234026	n/a	n/a
D26/Delmarva	2020	Delmarva	Sentinel	*	*	*	*	*	-	-	-	-	-	n/a	n/a	n/a	n/a	n/a	n/a	n/a
D27/Delmarva	2020	Delaware	Sentinel	*	*	*	*	*	+	+	+	+	+	-	-	-	PX233981	PX234023	n/a	n/a
D28/Delmarva	2020	Delaware	Sentinel	*	*	*	*	*	-	-	-	-	-	n/a	n/a	n/a	n/a	n/a	n/a	n/a
D29/Delmarva	2020	Delaware	Sentinel	*	*	*	*	*	+	+	+	-	+	-	-	-	n/a	PX234049	n/a	n/a
M1/Delmarva	2021	Maryland	Commercial	21	0.63	no	no	yes	+	-	+	-	-	-	-	-	n/a	n/a	n/a	n/a
M2/Delmarva	2021	Delaware	Commercial	18	0.30	no	*	yes	+	-	+	-	-	-	-	-	n/a	n/a	n/a	n/a
M3/Delmarva	2021	Virginia	Commercial	15	0.40	yes	no	yes	+	-	+	-	-	-	-	-	n/a	n/a	n/a	n/a
M4/Delmarva	2021	Delaware	Sentinel	*	*	*	*	*	+	-	+	-	-	-	-	-	n/a	n/a	n/a	n/a
M5/Delmarva	2021	Maryland	Commercial	21	0.50	no	yes	yes	-	-	-	-	-	n/a	n/a	n/a	n/a	n/a	n/a	n/a
M6/Delmarva	2021	Virginia	Commercial	12	0.40	no	yes	yes	+	-	+	-	-	-	-	-	n/a	n/a	n/a	n/a
M7/Delmarva	2021	Virginia	Commercial	24	0.30	no	yes	yes	+	+	+	+	+	-	-	-	n/a	PX234043	PX234009	n/a
M8/Delmarva	2021	Maryland	Commercial	24	0.44	yes	yes	yes	+	+	+	+	+	-	-	-	PX233970	PX234046	n/a	n/a
M9/Delmarva	2021	Maryland	Commercial	18	0.90	yes	yes	yes	+	-	+	-	-	-	-	-	n/a	n/a	n/a	n/a
M10/Delmarva	2021	Maryland	Commercial	17	1.00	yes	yes	yes	-	-	-	-	-	n/a	n/a	n/a	n/a	n/a	n/a	n/a
M11/Delmarva	2021	Maryland	Commercial	28	0.19	no	yes	yes	+	+	+	+	-	-	-	-	PX234004	n/a	n/a	n/a
M12/Delmarva	2021	Delaware	Commercial	18	0.57	yes	*	yes	+	-	+	-	-	-	-	-	n/a	n/a	n/a	n/a
M13/Delmarva	2021	Maryland	Commercial	18	1.20	no	yes	yes	+	-	+	-	-	-	-	-	n/a	n/a	n/a	n/a
M14/Delmarva	2021	Maryland	Commercial	22	1.10	yes	yes	yes	+	+	+	-	-	-	-	-	n/a	n/a	n/a	n/a
M15/Delmarva	2021	Delaware	Commercial	21	0.50	yes	yes	yes	+	+	+	+	+	-	-	-	PX233978	PX234032	n/a	n/a
M16/Delmarva	2021	Maryland	Commercial	28	0.18	yes	yes	yes	+	-	+	-	-	-	-	-	n/a	n/a	n/a	n/a
M17/Delmarva	2021	Maryland	Commercial	15	0.80	yes	*	yes	-	-	-	-	-	n/a	n/a	n/a	n/a	n/a	n/a	n/a
M18/Delmarva	2021	Maryland	Commercial	15	0.40	yes	yes	yes	+	-	+	-	-	-	-	-	n/a	n/a	n/a	n/a
M19/Delmarva	2021	Maryland	Commercial	28	0.60	yes	yes	yes	+	+	+	+	+	-	-	-	PX233979	PX234028	n/a	n/a
M20/Delmarva	2021	Virginia	Commercial	20	0.45	yes	yes	yes	-	-	-	-	-	n/a	n/a	n/a	n/a	n/a	n/a	n/a
M21/Delmarva	2021	Maryland	Commercial	19	0.53	yes	yes	yes	-	-	-	-	-	n/a	n/a	n/a	n/a	n/a	n/a	n/a
M22/Delmarva	2021	Maryland	Commercial	32	0.20	yes	yes	yes	+	+	-	-	-	-	-	-	n/a	n/a	n/a	n/a
M23/Delmarva	2021	Maryland	Commercial	28	0.40	yes	yes	yes	+	+	+	+	-	-	-	-	n/a	n/a	PX234018	n/a
M24/Delmarva	2021	Delaware	Commercial	18	0.80	yes	*	yes	-	-	-	-	-	n/a	n/a	n/a	n/a	n/a	n/a	n/a
M25/Delmarva	2021	Virginia	Commercial	24	0.55	yes	*	yes	+	+	-	+	-	-	-	-	n/a	n/a	PX234016	n/a
D48/Delmarva	2021	Delaware	Sentinel	*	*	*	*	*	+	+	+	+	+	-	-	-	n/a	n/a	PX234014	PX233334
D49/Delmarva	2021	Delaware	Sentinel	*	*	*	*	*	+	+	+	+	+	-	-	-	PX233973	PX234050	n/a	n/a
D50/Delmarva	2021	Delaware	Sentinel	*	*	*	*	*	-	-	-	-	-	n/a	n/a	n/a	n/a	n/a	n/a	n/a
D51/Delmarva	2021	Delaware	Sentinel	*	*	*	*	*	-	-	-	-	-	n/a	n/a	n/a	n/a	n/a	n/a	n/a
D52/Delmarva	2021	Delaware	Sentinel	*	*	*	*	*	-	-	-	-	-	n/a	n/a	n/a	n/a	n/a	n/a	n/a
D53/Delmarva	2021	Delaware	Sentinel	*	*	*	*	*	-	-	-	-	-	n/a	n/a	n/a	n/a	n/a	n/a	n/a
D54/Delmarva	2021	Delaware	Sentinel	*	*	*	*	*	-	-	-	-	-	n/a	n/a	n/a	n/a	n/a	n/a	n/a
D55/Delmarva	2021	Delmarva	Sentinel	*	*	*	*	*	+	+	+	+	+	-	-	-	n/a	n/a	PX234020	PX233336
D56/Delmarva	2021	Delmarva	Sentinel	*	*	*	*	*	+	+	+	+	+	-	-	-	PX233977	PX234033	n/a	n/a
D57/Delmarva	2021	Delmarva	Sentinel	*	*	*	*	*	-	-	-	-	-	n/a	n/a	n/a	n/a	n/a	n/a	n/a
D58/Delmarva	2021	Delmarva	Sentinel	*	*	*	*	*	+	+	-	+	-	-	-	-	PX233971	n/a	n/a	n/a
D59/Delmarva	2021	Delmarva	Sentinel	*	*	*	*	*	-	-	-	-	-	n/a	n/a	n/a	n/a	n/a	n/a	n/a
D60/Delmarva	2021	Delmarva	Sentinel	*	*	*	*	*	+	+	+	+	+	-	-	-	PX234001	PX234035	n/a	n/a
D61/Delmarva	2021	Delmarva	Sentinel	*	*	*	*	*	-	-	-	-	-	n/a	n/a	n/a	n/a	n/a	n/a	n/a
D62/Delmarva	2021	Delmarva	Sentinel	*	*	*	*	*	+	+	+	+	+	-	-	-	PX233995	PX234030	n/a	n/a
D63/Delmarva	2021	Delmarva	Sentinel	*	*	*	*	*	+	+	+	+	+	-	-	-	PX233994	PX234029	n/a	n/a
D64/Delmarva	2021	Delmarva	Sentinel	*	*	*	*	*	-	-	-	-	-	n/a	n/a	n/a	n/a	n/a	n/a	n/a
D65/Delmarva	2021	Delmarva	Sentinel	*	*	*	*	*	-	-	-	-	-	n/a	n/a	n/a	n/a	n/a	n/a	n/a
D66/Delmarva	2021	Delmarva	Sentinel	*	*	*	*	*	-	-	-	-	-	n/a	n/a	n/a	n/a	n/a	n/a	n/a
D67/Delmarva	2021	Delmarva	Sentinel	*	*	*	*	*	+	+	-	+	-	-	-	-	PX233992	n/a	n/a	n/a
D68/Delmarva	2021	Delmarva	Sentinel	*	*	*	*	*	-	-	-	-	-	n/a	n/a	n/a	n/a	n/a	n/a	n/a
D69/Delmarva	2021	Delmarva	Sentinel	*	*	*	*	*	-	-	-	-	-	n/a	n/a	n/a	n/a	n/a	n/a	n/a
D70/Delmarva	2021	Delaware	Sentinel	*	*	*	*	*	+	+	-	+	-	-	-	-	PX233975	n/a	n/a	n/a
D71/Delmarva	2021	Delaware	Sentinel	*	*	*	*	*	-	-	-	-	-	n/a	n/a	n/a	n/a	n/a	n/a	n/a
D72/Delmarva	2021	Delaware	Sentinel	*	*	*	*	*	+	+	-	+	-	-	-	-	PX233980	n/a	n/a	n/a
D73/Delmarva	2021	Delaware	Sentinel	*	*	*	*	*	-	-	-	-	-	n/a	n/a	n/a	n/a	n/a	n/a	n/a
D74/Delmarva	2021	Delaware	Sentinel	*	*	*	*	*	-	-	-	-	-	n/a	n/a	n/a	n/a	n/a	n/a	n/a
D75/Delmarva	2021	Delmarva	Sentinel	*	*	*	*	*	+	+	-	+	-	-	-	-	PX234005	n/a	n/a	n/a
D76/Delmarva	2021	Delmarva	Sentinel	*	*	*	*	*	+	+	+	+	+	-	-	-	n/a	n/a	PX234017	PX233333
D77/Delmarva	2021	Delmarva	Sentinel	*	*	*	*	*	+	+	-	+	-	-	-	-	PX233999	n/a	n/a	n/a
D78/Delmarva	2021	Delmarva	Sentinel	*	*	*	*	*	+	+	-	+	-	-	-	-	PX233996	n/a	n/a	n/a
M26/Delmarva	2022	Virginia	Commercial	25	0.43	yes	yes	yes	-	-	-	-	-	n/a	n/a	n/a	n/a	n/a	n/a	n/a
M27/Delmarva	2022	Delaware	Commercial	31	0.35	yes	yes	yes	-	-	-	-	-	n/a	n/a	n/a	n/a	n/a	n/a	n/a
M28/Delmarva	2022	Maryland	Commercial	18	0.40	yes	*	yes	+	+	+	+	+	-	-	-	PX233972	PX234036	n/a	n/a
M29/Delmarva	2022	Virginia	Commercial	35	0.30	yes	yes	yes	-	-	-	-	-	n/a	n/a	n/a	n/a	n/a	n/a	n/a
D88/Delmarva	2022	Delmarva	Sentinel	*	*	*	*	*	+	+	-	+	-	-	-	-	PX234000	n/a	n/a	n/a
D89/Delmarva	2022	Delmarva	Sentinel	*	*	*	*	*	+	+	-	+	-	-	-	-	PX233991	n/a	n/a	n/a
D90/Delmarva	2022	Delmarva	Sentinel	*	*	*	*	*	-	-	-	-	-	n/a	n/a	n/a	n/a	n/a	n/a	n/a
M30/Delmarva	2023	Delaware	Commercial	34	0.25	yes	yes	yes	+	+	+	+	+	-	-	-	PX233974	PX234034	n/a	n/a
M31/Delmarva	2023	Maryland	Commercial	24	0.35	yes	yes	yes	+	+	+	+	+	-	-	-	n/a	PX234024	PX234019	n/a
M32/Delmarva	2023	Maryland	Commercial	29	0.53	no	yes	yes	+	+	+	+	+	-	-	-	PX233986	PX234025	n/a	n/a
M33/Delmarva	2023	Virginia	Commercial	28	0.40	no	yes	yes	+	+	-	+	-	-	-	-	PX233984	n/a	n/a	n/a
M34/Delmarva	2023	Virginia	Commercial	34	0.75	yes	yes	yes	-	-	-	-	-	n/a	n/a	n/a	n/a	n/a	n/a	n/a
M35/Delmarva	2023	Virginia	Commercial	29	0.50	yes	yes	yes	+	+	+	+	-	-	-	-	PX233983	n/a	n/a	n/a
M36/Delmarva	2023	Virginia	Commercial	36	0.36	yes	yes	yes	+	+	+	+	-	-	-	-	PX233997	n/a	n/a	n/a
M37/Delmarva	2023	Maryland	Commercial	16	0.50	yes	yes	yes	-	-	-	-	-	n/a	n/a	n/a	n/a	n/a	n/a	n/a
M38/Delmarva	2023	Maryland	Commercial	29	0.30	no	yes	yes	+	+	-	+	-	-	-	-	PX234002	n/a	n/a	n/a
M39/Delmarva	2023	Maryland	Commercial	17	0.50	yes	yes	yes	+	+	-	+	-	-	-	-	PX234006	n/a	n/a	n/a
M40/Delmarva	2023	Delaware	Commercial	26	0.65	yes	yes	yes	-	-	-	-	-	n/a	n/a	n/a	n/a	n/a	n/a	n/a
M41/Delmarva	2023	Maryland	Commercial	17	0.30	yes	*	yes	-	-	-	-	-	n/a	n/a	n/a	n/a	n/a	n/a	n/a
M42/Delmarva	2023	Maryland	Commercial	20	0.20	yes	yes	yes	+	+	-	+	-	-	-	-	PX234007	n/a	n/a	n/a
M43/Delmarva	2023	Maryland	Commercial	21	0.30	yes	*	yes	-	-	-	-	-	n/a	n/a	n/a	n/a	n/a	n/a	n/a
M44/Delmarva	2023	Maryland	Backyard	**	*	no	*	yes	+	-	+	-	-	-	-	-	n/a	n/a	n/a	n/a
M45/Delmarva	2023	Maryland	Commercial	18	0.80	yes	yes	yes	+	+	-	-	-	-	-	-	n/a	n/a	n/a	n/a
M46/Delmarva	2023	Maryland	Commercial	17	0.70	yes	*	yes	-	-	-	-	-	n/a	n/a	n/a	n/a	n/a	n/a	n/a
M47/Delmarva	2023	Maryland	Commercial	18	0.90	yes	yes	yes	-	-	-	-	-	n/a	n/a	n/a	n/a	n/a	n/a	n/a
M48/Delmarva	2023	Delaware	Commercial	16	0.30	yes	yes	yes	-	-	-	-	-	n/a	n/a	n/a	n/a	n/a	n/a	n/a
M49/Delmarva	2023	Delaware	Commercial	23	0.30	yes	yes	yes	-	-	-	-	-	n/a	n/a	n/a	n/a	n/a	n/a	n/a
M50/Delmarva	2023	Virginia	Commercial	18	0.50	yes	*	yes	+	+	+	+	-	-	-	-	PX233990	n/a	n/a	n/a
D91/Delmarva	2023	Delmarva	Sentinel	*	*	*	*	*	+	+	+	+	+	-	-	-	PX233993	PX234044	n/a	n/a
D92/Delmarva	2023	Delmarva	Sentinel	*	*	*	*	*	+	+	+	+	+	-	-	-	PX233989	PX234042	n/a	n/a
D93/Delmarva	2023	Delmarva	Sentinel	*	*	*	*	*	+	+	+	+	+	-	-	-	n/a	n/a	PX234010	PX233335
D94/Delmarva	2023	Delmarva	Sentinel	*	*	*	*	*	+	+	+	+	+	-	-	-	PX233988	PX234040	n/a	n/a
D95/Delmarva	2023	Delmarva	Commercial	27	*	*	*	*	+	+	+	+	+	-	-	-	n/a	n/a	PX234013	PX233332
D96/Delmarva	2023	Delmarva	Commercial	23	*	*	*	*	+	+	+	+	+	-	-	-	PX234003	PX234051	n/a	n/a
D97/Delmarva	2023	Delmarva	Sentinel	*	*	*	*	*	+	+	+	+	+	-	-	-	PX233998	PX234038	n/a	n/a

* Information not available
** Backyard origin 9 months
n/a Not applicable

Table S1. Metadata, clinical findings, diagnostic results, and sequencing information for bursal samples collected in the Delmarva region from 2018 to 2023. Shown are sample identification, year and State of origin, bird type (sentinel, commercial, or backyard), age at sampling, reported daily mortality, and the presence of macroscopic and microscopic bursal lesions. RT-PCR results for infectious bursal disease virus (IBDV) targeting segment A (VP2 hypervariable region) and segment B (VP1), as well as RT-PCR screening for avian reovirus, avian rotavirus, and chicken astrovirus, are indicated. Both PCR-positive and PCR-negative samples are included, including clinically suspicious cases with compatible bursal lesions but negative molecular detection. Sequencing status (partial or whole VP2 and VP1) and corresponding GenBank accession numbers are provided.
