# Supplementary material for: Molecular characterization of infectious bursal disease virus (IBDV) strains of genogroup A2B1 circulating in Delaware, Maryland, and Virginia from 2018 to 2023
Source: Microbiol Spectr. 2026 Apr 16;14(6):e02976-25. doi: 10.1128/spectrum.02976-25 (PMC13227996; doi:10.1128/spectrum.02976-25)
Supplement: Supplemental figure legends [file spectrum.02976-25-s0008.docx]

**Molecular characterization of Infectious Bursal Disease Virus (IBDV) strains of genogroup A2B1 circulating in Delaware, Maryland and Virginia from 2018-2023.**

Sofia Egana-Labrin^1,2^, Andrew Brodrick^1^, Zubair Khalid^1^, Declan Kehlbeck^1^, Megan Liu^1^, Jimmy Dong^1^, Alex Broadway^1^, Milos Markis^3^, Shankar P. Mondal^4+^, Andrew J Broadbent^1^

^1^Department of Animal and Avian Sciences, University of Maryland, College Park, MD, 20742

^2^Poultry Research and Diagnostic Laboratory, College of Veterinary Medicine, Mississippi State University, Pearl, MS, 39288

^3^AviServe LLC, Newark, DE, 19702

^4^Salisbury Animal Health Laboratory Maryland Department of Agriculture, Salisbury, MD 21801

^+^Deceased

*Corresponding authors: sce114@msstate.edu; [ajbroad@umd.edu](mailto:venugopal.nair@pirbright.ac.uk)

**Supplemental Figures**

**Fig. S1. Phylogenetic tree and the corresponding root-to-tip divergence plots based on the HVR of the VP2 gene, focusing on US genogroup A2 clade 2 sub-clades. (A)** The phylogeny was inferred using the Maximum Likelihood method and Tamura-Nei (1993) model and the tree with the highest log likelihood is shown. Evolutionary analyses were conducted and visualized in MEGA12. The tree was constructed using 42 HVR nucleotide sequences obtained from Sanger sequencing of the PCR products and the nucleotide sequences of 3 reference IBDV Genogroup A2 Clade 2 US variant strains in GenBank (JF736000, JF736003, and JF736005). All the sequences were trimmed to nucleotides 649 to 1,125 of the VP2 gene, to be the same length. The tree was divided into eight sub-clades, each depicted by a bracket. **(B)** Individual root-to-tip divergence plots of the VP2 HVR Genogroup A2 Clade 2 sub-clades. The x-axis of the root-to-tip divergence plot shows the collection year, and the y-axis shows the root-to-tip divergence on the maximum-likelihood phylogenetic tree. The linear regression is represented by the black line of the root-to-tip divergence and collection year. All the strains with four or more sequences from different years available were analyzed.

**Fig. S2. Alignment of the VP2 HVR amino acid sequences from Delmarva in 2018-2023 compared to the Delaware E and AL-2.** The 53 nucleotide sequences were translated in silico and the amino acid sequences aligned and compared to US A2B1 strains Del-E (Accession number X54858) and AL-2 (Unpublished sequence, kindly provided by Dr. Milos Markis). Each amino acid was depicted by its letter abbreviation. Where the amino acid was identical to the reference strain, it was shown as a dot. The boxes indicate the HVR hydrophilic loops P-BC, P-DE, P-FG, and P-HI. The alignment was abbreviated by removing identical amino acid positions after the initial and before the last residue, to emphasize the amino acid differences compared to the reference strain. **(A)** Alignment compared to Delaware E strain, the amino acid substitutions that were observed in the majority of strains and therefore appeared in the consensus sequence were highlighted (S254N, green; S317R, purple; G322E, blue; E323D, red). **(B)** Alignment compared to AL-2 strain, the amino acid substitutions that were observed in the majority of strains and therefore appeared in the consensus sequence were highlighted (S254N, green; S317R, purple; N318D, violet; E321A, yellow; G322E, blue; E359T, turquoise).

**Fig. S3. Alignment of the full-length VP2 amino acid sequences from Delmarva in 2018-2023 compared to the Del-E variant.** The 15 nucleotide sequences spanning the entire VP2 coding region were translated in silico and the amino acid sequences aligned and compared to Del-E (Accession number X54858). Each amino acid was depicted by its letter abbreviation. Where the amino acid was identical to Del-E, it was shown as a dot. The boxed regions indicate the HVR hydrophilic loops P-BC, P-DE, P-FG, and P-HI, respectively. Amino acid substitutions that were observed in the majority of strains and therefore appeared in the consensus sequence were highlighted (S254N, green; S317R, purple; G322E, blue; E323D, red).

**Fig. S4. Alignment of the partial VP1 amino acid sequences from Delmarva in 2018-2023 compared to the Del-E variant.** The 34 nucleotide sequences spanning a 498 bp of length region of VP1 that were translated in silico and the amino acid sequences aligned and compared to Del-E (Accession number ON100678). Very virulent strains UK661 (Accession number NC004179) and rA California (Accession number GQ221684) were also included for comparison. Each amino acid was depicted by its letter abbreviation, and when the amino acid was identical to Del-E, it was shown as a dot. The box indicates the triplet of amino acids at positions 145-147 that are associated with IBDV virulence. The alignment was abbreviated by removing identical amino acid positions after the initial and before the last residue to emphasize the amino acid differences compared to Del-E. Amino acid substitutions within the triplet were highlighted orange/yellow, and amino acid substitutions outside of the triplet were highlighted grey.

**Fig. S5. Alignment of the full-length VP1 amino acid sequences from Delmarva compared to the Del-E variant.** The 5 nucleotide sequences spanning the entire VP1 coding region were translated in silico and the amino acid sequences aligned and compared to Del-E (Accession number ON100678). Each amino acid was depicted by its letter abbreviation. Where the amino acid was identical to Del-E, it was shown as a dot. The box indicates the triplet of amino acids at positions 145-147 that are associated with IBDV virulence. Amino acid substitutions within the triplet were highlighted orange/yellow, and amino acid substitutions outside of the triplet were highlighted grey.

**Fig. S6.** **Structural modelling of the VP1 protein.** The predicted structure of the Del-E VP1 polymerase protein was modelled using AlphaFold3. The predicted structure was loaded and visualized with PyMol. **(A)** The catalytic cleft of the polymerase was depicted in the center of the molecule, the Del- E atoms were depicted in grey and the positions of the amino acid triplet at residues 145-147 were mapped onto the structure and highlighted orange. The genetic signature comprised of amino acid substitutions I385V, R508K, K579R, R682K, and S718N that were present in 4/5 (80%) of full-length VP1 sequences were highlighted blue. **(B)** The same structure flipped 180º on its horizontal axis.
